# Identification and Evolution of Core Technologies in the Chip Field Based on Patent Networks

**DOI:** 10.3390/e27060617

**Published:** 2025-06-10

**Authors:** Ying Wang, Renda Chen, Jindong Chen

**Affiliations:** Management Science and Engineering College, Beijing Information Science and Technology University, Beijing 102206, China; bistu_wy@126.com (Y.W.); dapengu127@163.com (R.C.)

**Keywords:** chip, patent network, information entropy, SPNP Main Path, technological evolution

## Abstract

Currently, the global technological competition pattern is accelerating its restructuring, and chip technology, as a core technology for national strategic security and industrial competition, faces a serious bottleneck that seriously restricts the construction of China’s industrial chain security and innovation ecology. A “recognition-evolution” collaborative analysis system was proposed in this study using patent data as a carrier. Firstly, a PKCN-BERT-LDA fusion module was constructed to identify the core technologies of chip design, manufacturing, and packaging testing. Secondly, the traditional main path analysis method was improved by innovatively introducing information entropy theory to construct a dynamic evolution model, and the technological evolution path in the chip field during 2010–2024 was systematically tracked based on the Derwent patent database. According to this study, the field of chip design exhibited a bidirectional innovation feature of “system optimization regional deep cultivation”, while the manufacturing process highlights the non-linear accumulation law of process complexity. Packaging and testing technology tended to develop in synergy with integration and intelligence.

## 1. Introduction

From the EU’s Horizon 2020 to Germany’s Industry 4.0 Strategic Plan, and to China’s 14th Five-Year National Science and Technology Innovation Plan, countries around the world are actively formulating science and technology development strategies and strengthening innovation deployment. The breakthrough of core technology has become a core proposition related to national strategic security and industrial competition patterns. As the underlying architecture of the digital economy and the core support of the modern industrial system, chip technology has risen to a critical dimension of national competition in terms of strategic importance. Currently, the global chip industry presented a “dual imbalance” pattern: on the one hand, advanced process technology is highly concentrated in a few international giants, forming a “technology lock-in” effect; on the other hand, China has significant external dependence in key areas such as EDA tools and semiconductor materials. This “bottleneck” dilemma not only restricts the upgrading of strategic emerging industries but also may trigger the risk of “chain breakage” in the industrial chain. Thus, it requires us to establish a collaborative framework for core technology identification and evolutionary analysis of the chip field. This “identification evolution” composite analysis system can not only help technology decision-makers clarify the focus of technological competition but also provide strategic predictions for the path evolution of technological breakthroughs, which has a dual support role in solving the “bottleneck” problems in the chip field in China.

As the core carrier of technological innovation, patents directly reflect the level of industrial technological development and competitive advantages. According to data from the World Intellectual Property Office [1], approximately 90–95% of global inventions were first publicly disseminated through patent documents. The detailed technical details and strong availability of patent data made them a strategic resource for technological competition.

Recent research on patent networks has centered on three areas: citation networks, co-occurrence networks, and cooperation networks. Studies on patent citation networks focus on value assessment, technology forecasting, and trend analysis. Key contributions include Yang et al., who developed a Comprehensive Patent Citation (CPC) network model for patent valuation [2]; Ji et al., analyzing dynamic evolution patterns in information technology [3]; and Zhu et al., identifying critical innovation paths through data integration [4]. Further advancements involve Pan et al.’s disruptive patent identification using regression models [5], Sun et al.’s time-enhanced SPC algorithm for fuel cell technology evolution [6], and Wang et al.’s hybrid main-derivative path method for tracking technological trajectories [7]. These works collectively enhance analytical frameworks through algorithmic innovation and multidimensional data integration.

Current core technology identification methods primarily fall into three categories: patent quantitative index evaluation systems, patent network topology analysis models, and multi-source data fusion frameworks. In patent quantitative evaluation, scholars often equate core patents with technological competitiveness. Examples include Tian Xuejiao et al.’s 3D framework integrating technology price, market distribution, and legal protection using entropy-weighted TOPSIS [8], and Wang et al.’s wind power technology model incorporating patent litigation participation and claim semantic breadth [9]. Network science has enabled patent topology analysis as a key paradigm. Mao Jianqi et al. developed an IPC co-classification topology framework to reveal cross-domain core correlations through module co-occurrence and community structures [10]. Qi Yun et al. combined small-world networks with dynamic citation analysis to track knowledge diffusion paths [11]. Multi-source data fusion frameworks leverage big data and AI. Yang Feng mapped technology transfer paths via LDA topic clustering and country maturity curves [12]. Xu Zonghuang et al. fused structural hole theory, LDA topic strength, and patent family data for lithography barrier analysis [13]. Yang Heng et al. employed entropy-weighted TOPSIS and Word2Vec mapping for phased AI patent screening [14].

Current technological evolution analysis methods are broadly categorized into citation network main path extraction, text topic mining, and hybrid fusion approaches. Main path extraction leverages citation networks to trace technological trajectories. Bhatt employed critical path analysis on blockchain patents to delineate disruptive innovation stages [15]; Kumar et al. mapped mobile payment evolution through citation networks and validated results with real-world cases [16]; Oh et al. proposed a refined main path identification framework for patent literature [17]. Text mining methods focus on implicit topic associations. Hu Zewen et al. fused BERT, LDA, and Word2Vec to model blockchain topic evolution, introducing a differentiation formula for emerging vs. hot technologies [18]; Xue et al. combined text vector reduction and N-gram mining to decode hydrogen energy recombination patterns [19]. Hybrid approaches integrate network and semantic insights. Suoqi et al. developed a multi-dimensional framework for measuring emerging industry evolution, enabling trajectory visualization [20]; Feng Lijie et al. synergized citation networks with SAO semantic analysis to enhance innovation path identification for enterprises [21].

Overall, there is still a significant void in academic research on the core technology system in the chip field, mainly manifested in the lack of systematic research paradigms and insufficient methodological innovation. The existing literature mostly focuses on static identification of single technology nodes or local links in the industrial chain, such as exploring the development path of photolithography technology [22,23]. There is a lack of collaborative evolution analysis of the entire chip industry chain technology ecosystem. Research on technological evolution often focuses on evolutionary paths, and the main path analysis method is commonly used to extract citations and analyze the process of technological evolution. Although this method can clearly demonstrate the trajectory of technological development. However, this method overly relies on the static topology structure of the patent citation network to identify the technological evolution path, which can easily overlook the technical content characteristics and dynamic uncertainty, resulting in overestimation of nodes with high citation but low innovation; in contrast, by introducing the theory of information entropy and combining the semantic features of technical content with citation relationships, dynamic quantification of the uncertainty of technological evolution can not only more accurately identify highly innovative core nodes, but also judge the turning point of technological trends through entropy fluctuations. In response to the limitations of existing research, the chip field was divided into three core stages: Chip design, manufacturing, and packaging testing. Taking the chip design process as an example, a core technology “recognition evolution” composite analysis framework was constructed: firstly, a graph neural network was used to fuse the semantic and structural features of patent text to generate vector representations, and density clustering technology was used to divide clusters; secondly, based on the topic model, core technology topics within the cluster were mined, and core nodes were screened through network analysis; subsequently, the patent retrieval formula was reconstructed to obtain the core technology patent dataset, and, finally, combined with dynamic information entropy features, the main path analysis method was used to reveal the technological evolution trajectory.

## 2. Materials and Methods

### 2.1. Research Methods and Processes for Core Technology Identification

#### 2.1.1. Dataset Collection

The Derwent Innovation Index (DII) integrates the value-added data from the Derwent World Patents Index (DWPI), covering inventions from over 100 patent-granting authorities worldwide. Its scope is regarded as representing more than 96% of publicly available patent technologies globally. This nearly comprehensive coverage serves as a foundational assurance that research conclusions are not confined to specific countries or regions, providing a global perspective and universality. The core value of DWPI lies in its professional integration of “patent families” and manual indexing. Derwent’s technical experts consolidate patents for the same invention filed in different countries (patent families) into a single record, eliminating duplicate counts and providing a more accurate reflection of an invention’s global landscape and true value. Furthermore, the patent citation data from the Derwent Patents Citation Index (DPCI) constructs a robust technical association network. The DPCI includes not only the literature cited by patent examiners (ensuring legal relevance) but also the literature referenced by inventors (revealing technological relevance) [24]. This multidimensional citation information is crucial for identifying core technologies, tracking knowledge flows, and predicting trends in technological development [25].

Based on these advantages, this study selects the Derwent Innovation Index (DII) as the source of patent data. This study focuses on the chip field, with data collection covering chip-related patents published on the DII platform from 2010 to 2024. The specific retrieval time window was set from 1 January 2010 to 31 December 2024.

This study developed the initial patent search query by incorporating integrated circuit-related search strategies [26] and referencing chip-related technical terminology from the “Patent Classification System for Key Digital Technologies (2023)” issued by the China National Intellectual Property Administration (CNIPA). The search query was formulated as follows:

(1) Chip Design Search Query:

(TS = (“design” OR “simulate” OR “verify” OR “layout” OR “synthesis” OR “placement” OR “routing” OR “floorplan” OR “timing analysis”)) AND (TS = (“Electronic Design Automation” OR “EDA” OR “Electronic design automation” OR “circuit simulate*” OR “FPGA design*” OR “circuit layout” OR “IC design*” OR “IC simulation” OR “chip simulate*” OR “chip design*” OR “Intellectual Property core” OR “IP core” OR “ASIC design*” OR “RTL design*” OR “physical design” OR “SoC design*” OR “HDL” OR “Verilog” OR “VHDL”))

(2) Chip Manufacturing Search Query:

(TI = (clean OR lithograph OR photolithography OR electroplate OR deposition OR “film deposition” OR implant OR “ion implantation” OR anneal OR “laser annealing” OR epitaxy OR “epitaxial growth” OR oxidation OR patterning OR polishing OR cutting OR removing OR stripping OR etching OR “plasma etching” OR “dry etching” OR “wet etching” OR metallization OR thinning OR CVD OR PVD OR ALD OR ALE OR CMP OR “chemical mechanical planarization” OR transistor OR sputtering OR “etch stop layer”)) AND (AB = ((“integrated circuit”) OR IC OR “integrated Circuit” OR chip OR chipset OR microcircuit OR microchip OR semiconductor OR wafer OR die OR “substrate” OR “semiconductor material” OR transistor OR FinFET OR GAA OR “high-k dielectric” OR “low-k dielectric” OR “gate oxide”)) NOT (TI = (LED OR “light emitting diode” OR display OR “organic light emitting diode” OR OLED OR “quantum dot” OR “solar cell” OR photovoltaic OR “optoelectronic device” OR “laser diode” OR “light source”)) NOT (AB = (LED OR “light emitting diode” OR display OR “organic light emitting diode” OR OLED OR “quantum dot” OR “solar cell” OR photovoltaic OR “optoelectronic device” OR “laser diode” OR “light source”))

(3) Chip Packaging and Testing Search Query:

(TI = (package OR packaging OR “advanced packaging” OR interconnect OR bonding OR “wire bonding” OR TSV OR Mold OR “Through-Hole Mount” OR THM OR SOP OR QFP OR BGA OR “flip-chip” OR WLCSP OR “fan-out” OR test OR testing OR “reliability test” OR ATE)) AND (TS = (“integrated circuit” OR IC OR chip OR chipset OR microcircuit OR microchip OR “semiconductor device” OR “semiconductor package” OR semiconductor OR wafer OR SoC OR “stacked die” OR “electronic module” OR “module packaging”))

Given the massive volume of patent data and the relatively small proportion of core technologies within the dataset, patents with a citation frequency of five or above were selected as the research data. The data collection yielded 12,548, 18,458, and 22,716 patent records for chip design, chip manufacturing, and chip packaging and testing domains, respectively.

#### 2.1.2. Research Methods for Core Technology Identification

(1) BERT Model

**Figure 1:**
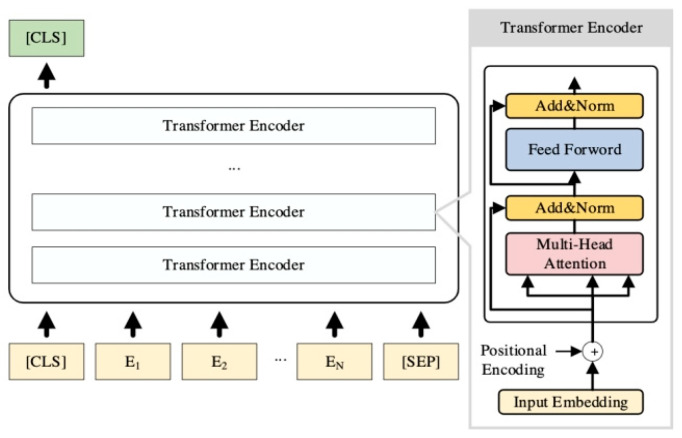
BERT Model.

BERT is a pre-trained language model launched by Google, as shown in Figure 1, which has achieved significant breakthroughs in the field of natural language processing. This model aims to overcome the unidirectionality limitation of traditional language models. Traditional models can only make unidirectional predictions (from left to right or from right to left) when processing text and cannot fully capture global contextual information [27]. BERT significantly improves its semantic understanding and feature extraction capabilities by adopting a bidirectional context encoding mechanism. However, in the field of patents, BERT faces specific challenges: the pre-training corpus may lack sufficient representation of specialized technical language. For highly specialized patent texts, domain-adaptive fine-tuning is necessary to reduce misunderstandings related to technical terms and complex expressions.

(2) GCN

Graph Convolutional Networks (GCNs) are a type of graph embedding learning algorithm proposed by Kipf et al. in 2017 [28]. The relationships between nodes can be captured by performing convolution operations to learn and represent the features of each node and its neighboring nodes in the network. All convolutional layers in the GCN model share information, which helps to generate high-quality embedded representations. It is currently widely used in tasks such as topic prediction [29] and text classification [30,31].

The GCN model generated embedded representations of nodes by accepting the node feature matrix X and adjacency matrix A. Therefore, it can effectively integrate heterogeneous data, such as text semantic information and citation relationship information of patents [32], infer potential relationships between patents, more comprehensively capture patent features, and improve the accuracy and comprehensiveness of patent vector representation.

The propagation process between neural network layers is shown in Formula (1), where $\tilde{A}$ = A + I, the degree matrix, σ(.) is the activation function, and H(0) is the node feature matrix X.

(1) $$H^{\left(l+1\right)}=\sigma \left({\tilde{D}}^{-\frac{1}{2}}\tilde{A}{\tilde{D}}^{-\frac{1}{2}}H^{\left(l\right)}W^{\left(l\right)}\right),$$

However, Graph Convolutional Networks (GCNs) encounter their own challenges when dealing with patent networks: when the number of layers exceeds two, the problem of over-smoothing may arise. In sparse patent networks, the effectiveness of feature learning can be limited, and handling dynamic changes in patent network structures becomes difficult.

(3) LDA Topic Model

Latent Dirichlet Allocation (LDA) is a text clustering method based on probabilistic generative models, proposed by BLEI et al. in 2003. This model infers the underlying thematic structure in the text collection by mining the co-occurrence patterns of words in the document. To address the common issue of overfitting in traditional topic models, LDA introduced the Hidden Dirichlet Distribution, modeling the distribution of topics and vocabulary as probability distributions, thereby enhancing the model’s generalization ability. LDA is essentially a three-layer Bayesian probability model, with a hierarchical structure consisting of a document layer, a topic layer, and a vocabulary layer. Through this hierarchical modeling approach, it can more accurately capture the semantic information of text.

Despite this, LDA has its limitations in processing patent data: the number of topics must be predetermined, and an improper choice can adversely affect the results. LDA may not perform well on short texts and patent abstracts that are dense with specialized terminology, and it fails to adequately capture subtle semantic differences in patent texts.

**Figure 2:**
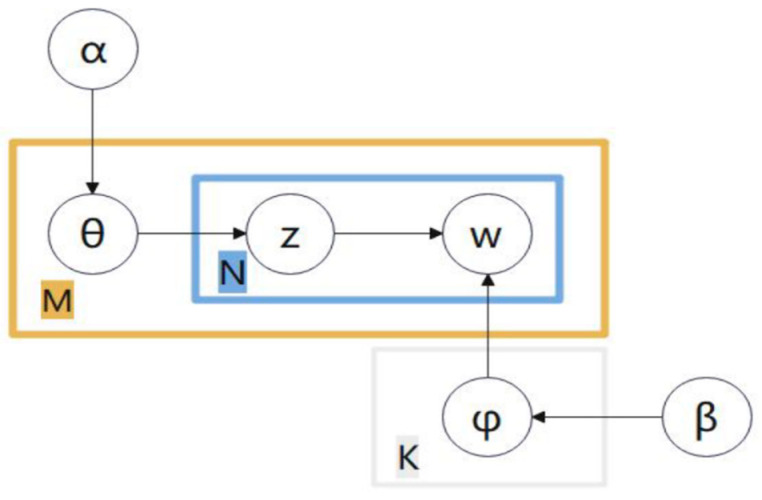
LDA Topic Model.

The “document-term” generation process of the LDA topic model, as shown in Figure 2, consists of the following steps: First, we sample the document-topic distribution θ from a Dirichlet distribution parameterized by α. Next, based on the document-topic distribution θ, we extract a topic Z for a specific word. Then, we select the topic-word distribution φ corresponding to topic Z from a Dirichlet distribution parameterized by β. Finally, we draw the word W according to the topic-word distribution φ. By iterating through this process multiple times, we can generate the “document-term” structure of a document. In this model, K represents the number of topics, box M indicates the iterative sampling process of the topic probability distribution for each document in the document collection, and box N represents the iterative process of drawing vocabulary for the document from the topic distribution.

#### 2.1.3. Research Processes for Core Technology Identification

Based on the above model, Python 3.7 was used for patent abstract text extraction and SpaCy for tagging to extract compound phrases of 2–4 words in the “noun + noun” and “verb + noun” patterns. Etymology was used to standardize word inflection and ensure standardized text input.

**Figure 3:**
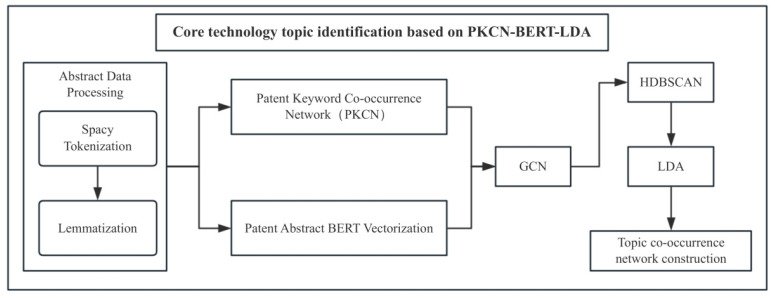
Flowchart of core technology identification research.

As shown in Figure 3, this study adopted a hybrid clustering method that combines text semantics and keyword network topology to improve clustering efficiency. The workflow consists of three stages:

(1) Construction of co-occurrence network: Python was used to build a patent keyword co-occurrence network and stored in the form of an adjacency table;
(2) Semantic vectorization: The BERT model generated 768-dimensional feature vectors for all patent abstracts;
(3) Multimodal fusion: Graph Convolutional Networks (GCNs) merge keyword network topology with text vectors was used to generate refined 768-dimensional representations for each patent.

For clustering analysis, HDBSCAN density clustering was initially applied. The post analysis revealed the dispersion within the cluster, where the main cluster contained multiple sub-clusters. LDA topic modeling was introduced for semantic refinement to address this issue. The core technology topic was determined through the following methods:

(1) Topic coherence optimization: The potential topic with the highest coherence score was selected;
(2) Topic co-occurrence matrix: It was constructed using documents with a topic probability greater than 0.1;
(3) Social network analysis: Core topics were determined through node centrality indicators and designate the top-ranked topics as core technologies.

### 2.2. Research Methods and Processes for Core Technology Evolution

#### 2.2.1. Patent Search Optimization

After identifying the core technologies, this study redesigned the patent search strategies based on the LDA core topic terms and the previously established search formulations, subsequently executing domain-specific retrieval queries to ensure comprehensive coverage of patents related to the target core technologies. The temporal scope of the search remained consistent with the aforementioned parameters.

#### 2.2.2. Research Methods for Core Technology Evolution

(1) SPNP Main Path Method

The SPNP method adopted the principle of node pair interaction, and the connection strength was evaluated by quantifying the co-occurrence probability of nodes at both ends of the edge in the path, where each node has dual attributes of information source point and aggregation point. Stronger discrimination was demonstrated in the identification of technological evolution paths by calculating the product relationship of path node pairs [33]. This method assigns higher weight values to intermediate nodes and connecting edges, effectively highlighting key breakthrough nodes and their transmission paths in the process of technological development. Based on this algorithm characteristic, SPNP was chosen as the criterion for determining the main path of technological evolution, to improve the accuracy of identifying the trajectory of core technological innovation.

**Figure 4:**
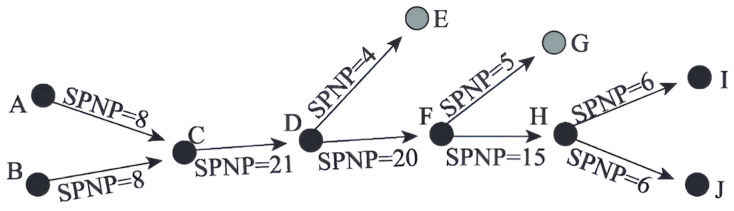
Fundamental principles of the SPNP algorithm.

As mentioned earlier, SPNP is the number of node pairs, mainly composed $\begin{array}{c} D_{vu} \end{array}$ of upstream and downstream nodes connected by directed edges, denoted as SPNP ($\begin{array}{c} D_{vu} \end{array}$):

(2) $$SPNP\left(\begin{array}{c} D_{vu} \end{array}\right)=L^{-}\left(u\right)\times L^{+}\left(v\right),$$

where $L^{-}\left(u\right)$ represented the u number of nodes cited by the patent, including u the patent itself and all nodes directly or indirectly cited by the patent; $L^{+}\left(v\right)$ indicates the number of nodes v that cite patents, including the v number of nodes that cite the patent itself and all directly or indirectly cited patents [34]. The SPNP value of each node was calculated, and, if the obtained SPNP statistical value tends to be larger, it represented a higher degree of importance in the connected network. The basic principles of the SPNP algorithm are shown in Figure 4.

At present, the main search methods include local main path, global main path, and critical path search. In response to the limitations of traditional main path analysis methods (forward and backward local tracking) in covering high SPNP value links, Liu et al. [35] proposed a critical path analysis method (Key-route). This method adopted a dual perspective deconstruction technique of global and local evolution path: firstly, a filtering mechanism based on link traversal weight extremum was established to determine the most influential core connections in the network; subsequently, the global critical path was constructed by incorporating the core link and maximizing the path weight integral function; the local critical path adopted a bidirectional expansion strategy, extending the path from the core link to the network source and sink points, and, finally, forming a complete technical transmission channel through topological merging.

(2) Information entropy

In thermodynamic systems, entropy is a physical quantity that describes the degree of disorder in the motion of microscopic particles, and its numerical characteristics depend only on the starting and ending states of the system. This characteristic made entropy an important indicator for measuring the degree of system chaos and stability, and its mathematical expression is shown in Formula (3). It is worth noting that entropy has the property of being independent of the observation order and always satisfies the non-negative condition, as shown in Formula (4).

(3) $$H=-k\sum_{i=1}^{p}\;P(x_{i}){log}_{E}P(x_{i}),$$

(4) $$H\left(x_{1},x_{2},\cdots ,x_{n}\right)=H\left(x_{n-2},x_{n-1},x_{n},\cdots ,x_{1}\right)⩾0,$$

where k represents any constant, usually with a default value of 1; p represents the total number of possible system and meet the requirements; $\sum_{i=1}^{n}\;P(x_{i})=1$; E is the base number, is the unit of entropy value: when E = 2, the unit is Bit, when E = e, unit is Nat, and when E = 10, the unitis Hart. $P(x_{i})$ Indicates the probability of $x_{i}$ a random event occurring.

#### 2.2.3. Research Processes for Core Technology Evolution

**Figure 5:**
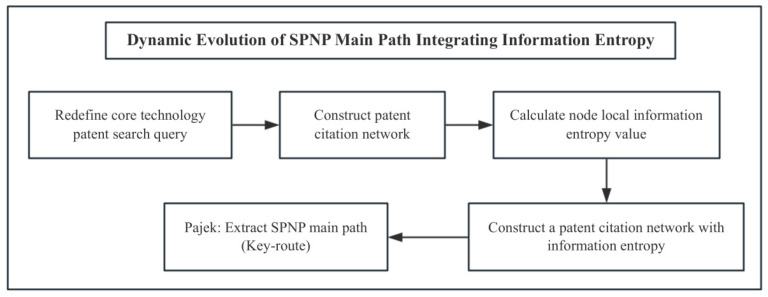
Flowchart of core technology evolution research.

Based on the above model, as shown in Figure 5, a four-stage analysis process was constructed:

(1) Local entropy calculation: Based on Python algorithms, the local entropy measure of each node in the citation network was calculated, quantifying the information uncertainty in technical knowledge diffusion;
(2) Key path extraction by integrating entropy: The Pajek network analysis tool extracted the key main paths through collaborative topological connectivity and entropy-weighted node saliency;
(3) Visualization of evolutionary trends: The trajectory of technological progress can be systematically characterized by mapping the entropy distribution pattern to node position attributes through a time trend chart.

## 3. Results

### 3.1. Analysis of Core Technology Identification Results in the Chip Field

#### 3.1.1. Identification of Core Technologies in Chip Design

**Figure 6:**
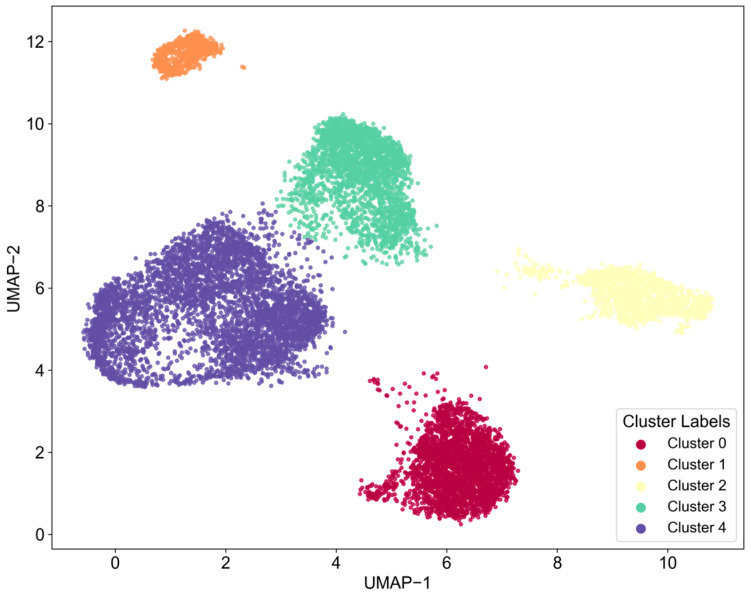
Chip design HDBSCAN clustering results.

**Figure 7:**
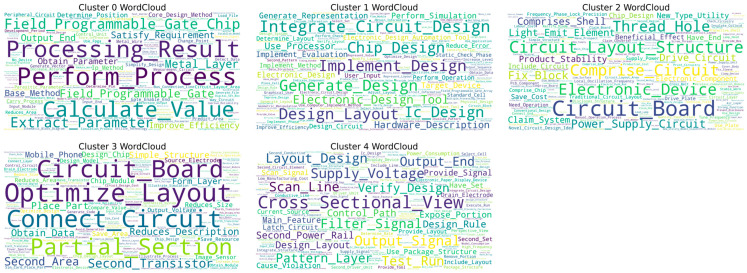
Chip design HDBSCAN clustering word cloud.

This study utilizes the HDBSCAN algorithm to cluster text data within the field of chip design, initially forming five distinct clusters (Cluster 0–4), as illustrated in Figure 6. The word cloud visualizations show that each cluster displays a certain thematic inclination, as detailed in Figure 7. High-frequency terms in Cluster 0, such as “Field Programmable Gate Chip” and “Optimize Design,” indicate a focus on programmable logic device design and optimization. In Cluster 1, terms like “Generate Design” suggest themes of design automation and generation. Clusters 2 and 3 prominently feature “Circuit_Layout,” accompanied by terms such as “Optimize Layout,” “Structure,” and “Power Supply Circuit,” emphasizing the physical layout of circuits and their optimization and highlighting the central importance of this theme within the dataset. Cluster 4 centers on keywords like “Circuit_Diagram” and “Semiconductor View,” focusing on the representation of circuit layers and device views.

However, a deeper analysis of the word cloud content reveals significant limitations in the current clustering results regarding the elucidation of core technical details. Firstly, the differentiation between themes is inadequate; for instance, common high-frequency terms such as “Design Rule,” “Schematic View,” and “Optimize Layout” appear redundantly across multiple clusters (e.g., Clusters 0, 2, and 3). This indicates that the algorithm may not have effectively disentangled foundational common concepts, leading to blurred boundaries between clusters and dilution of core technical features. Secondly, there is a lack of refined technical breakdowns within core themes, particularly evident in the critical area of circuit layout (Clusters 2 and 3). The word cloud only generally presents macro terms like “Circuit_Layout” and “Optimize Layout,” failing to distinguish between various types such as Analog Layout, Digital Place & Route, and RF Layout. It also lacks explicit representations of specific layout optimization techniques (e.g., timing-driven, power-driven, area optimization) or key metrics (e.g., routing congestion, signal integrity). Similarly, themes like design generation (Cluster 1) and circuit diagrams (Cluster 4) remain at a relatively general level.

Thus, while the clustering provides a preliminary outline of research hotspots, the existing results struggle to accurately map to specific, actionable core technical branches or methodological differences. To clearly define the characteristics of different technical routes and their corresponding relationships in classification, further refined analysis in conjunction with fine-grained feature engineering is essential.

Next, this study will focus on each major category from a fine-grained perspective, using LDA topic models and topic co-occurrence networks to identify the core technology topics within each major category.

Firstly, the coherence score was used to determine the number of topic divisions for each major category. The higher the coherence score, the more reasonable the topic division. The test results indicated that the number of topics in the clusters from 0 to 5 is 5, 7, 7, 7, and 6, respectively. Subsequently, the following results are obtained by analyzing the co-occurrence of topics in each major category and calculating the average degree of topic nodes, as shown in Table 1.

Through keyword analysis of each topic, five core technological topics were identified. Given the highly specialized nature of the semiconductor field, detailed explanations of each core technology are provided to facilitate reader comprehension, as presented in Table 2.

#### 3.1.2. Identification of Core Technologies in Chip Manufacturing

**Figure 8:**
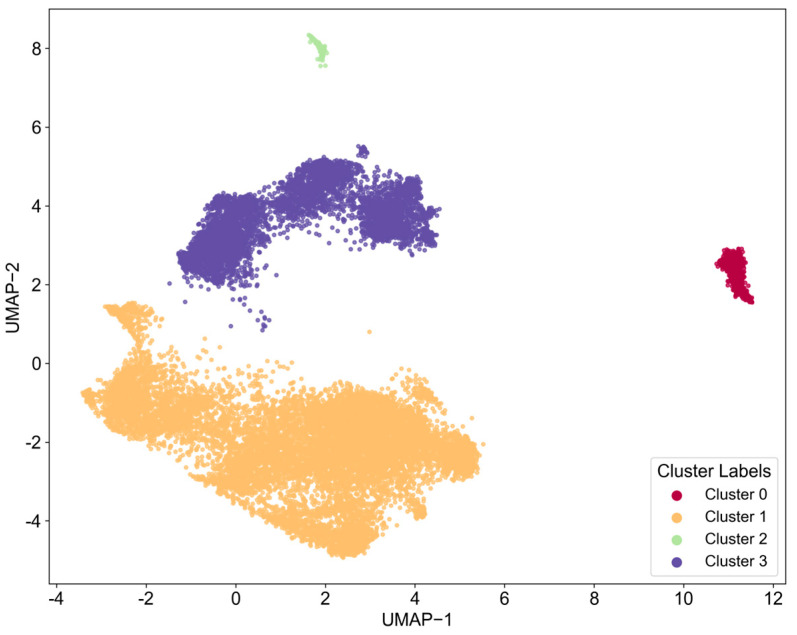
Chip manufacturing HDBSCAN clustering results.

**Figure 9:**
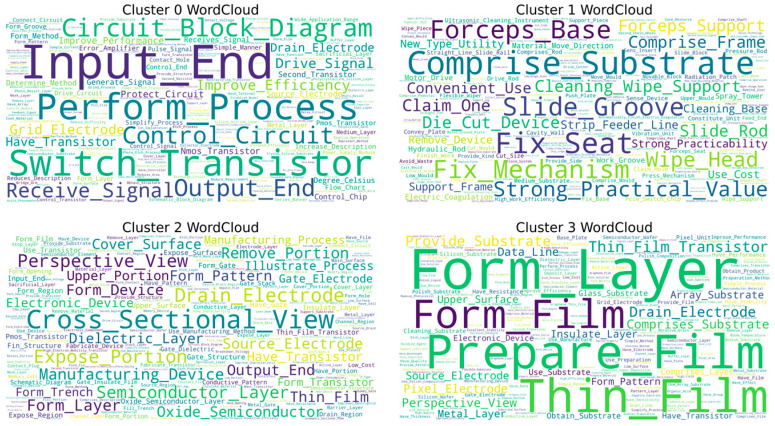
Chip manufacturing HDBSCAN clustering word cloud.

As shown in Figure 8 and Figure 9, the four word cloud clusters (Cluster 0–3) generated from text in the field of chip manufacturing using the HDBSCAN algorithm provide an initial insight into the differences in thematic distribution. Cluster 0 features high-frequency terms such as “Switch_Transistor,” “Circuit_Block_Diagram,” and “Protect_Circuit,” indicating a focus on transistor-level circuit design and protection mechanisms. In contrast, Cluster 1 is dominated by terms related to engineering support tools, such as “Fix_Mechanism,” “Cleaning_Wipe_Support,” and “Spray_Towel,” which significantly deviate from the core technical domain of chip manufacturing. Clusters 2 and 3 both involve view representations (“Schematic_View,” “Cross_sectional_View”) and process steps (“Form_Structure,” “Manufacturing_Method”), but they exhibit a high degree of overlap in core terms, with words like “Semiconductor_Device,” “Drain_Electrode,” and “Thin_Film” repeated across both clusters.

However, similar to the findings in the chip design sector, the current clustering results demonstrate clear deficiencies in identifying core technical features. Firstly, key technological elements are overly generalized, particularly within the core process clusters (Clusters 0, 2, and 3). For instance, high-frequency terms like “Thin_Film” and “Form_Structure” may point toward critical processes such as thin film deposition and structural formation, but they lack differentiation regarding specific technical pathways (e.g., CVD/PVD deposition methods, photolithography etching parameters) or material systems (e.g., high-k dielectrics, III-V compounds). Secondly, there is a noticeable disconnect in the core process chain; the word cloud fails to cluster significant manufacturing processes such as “Lithography,” “Etching,” and “Ion_Implantation.” Additionally, discrete device terms like “Drain_Electrode” and “Insulate_Line” appear in isolation, preventing the formation of a coherent sequence of process flows.

In summary, while the current classification exhibits surface-level thematic dispersion, it is constrained by a lack of technical granularity and missing interprocess connections, resulting in an inability to accurately map the core stages in chip manufacturing that have clear technological barriers. Therefore, it is essential to repeat the aforementioned steps to conduct a more detailed analysis.

Firstly, it is necessary to determine the number of LDA topics in each technology cluster. Based on the corresponding topic coherence scores, the number of topics is determined to be 17, 10, 16, and 12, respectively.

Subsequently, a document topic co-occurrence analysis was conducted on the topics of each technology cluster. In the analysis of technology cluster 0 and technology cluster 1, regardless of adjusting the threshold of document topic probability, the average value of all topic degrees within these two clusters remains consistent. Based on the number of patents in these two technology clusters, it could be inferred that this phenomenon is mainly due to the relatively insufficient number of patents: technology cluster 0 contains 893 patents, while technology cluster 1 only has 233 patents.

In view of this, the authors of this study believe that the topics within these two clusters are equally important, and there may be interrelationships between the topics within the same technology cluster. Therefore, it is reasonable to directly consider technology clusters 0 and 1 as two core technology topics. The analysis of technology clusters 2 and 3 is consistent with the previous method. The results are shown in Table 3.

Through LDA keyword analysis of each topic, four core technological topics were identified, as shown in Table 4.

#### 3.1.3. Identification of Core Technologies for Chip Packaging and Testing

**Figure 10:**
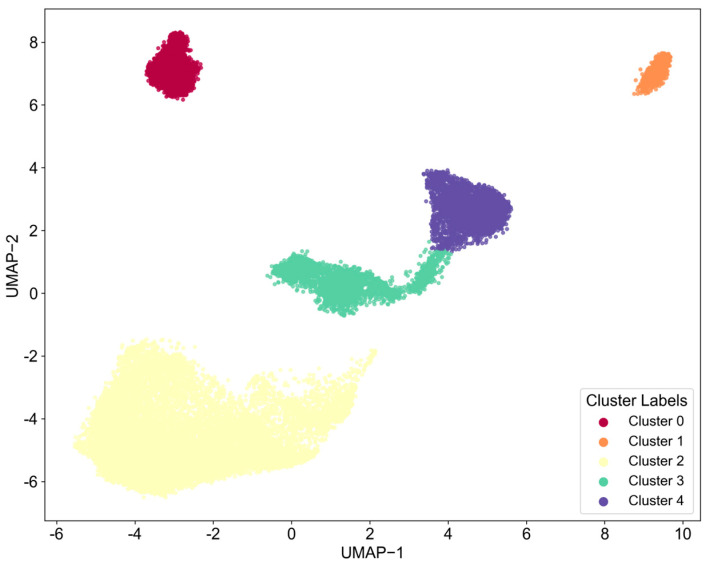
Chip packaging and testing HDBSCAN clustering results.

**Figure 11:**
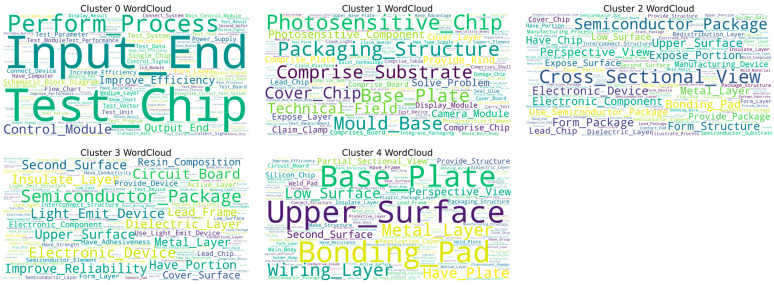
Chip packaging and testing HDBSCAN clustering word cloud.

As shown in Figure 10, the HDBSCAN algorithm has classified chip packaging and testing patent data into five clusters (Cluster 0–4). As shown in Figure 11, the word cloud visualizations indicate an initial differentiation of themes: Cluster 0 features high-frequency terms, such as “Test_Chip,” “Control_Module,” and “Test_Parameter,” which point towards testing systems and process control. Cluster 1 centers on key terms like “Comprise_Substrate” and “Photosensitive_Chip,” linking substrate materials to optoelectronic device packaging. Cluster 2 prominently includes terms like “Perspective_View,” “Cross_Sectional_View,” and “Semiconductor_Package,” highlighting the visual representation of packaging structures. Cluster 3 focuses on terms such as “Insulate_Layers,” “Light_Emit_Device,” and “Dielectric_Layer,” which relate to the integration of insulating materials and light-emitting devices. Finally, Cluster 4 revolves around terms like “Base_Plate,” “Wiring_Layer,” and “Bonding_Pad,” emphasizing substrate design and interconnect wiring.

Nevertheless, the current classification exhibits certain shortcomings in representing the core technologies of packaging and testing. Firstly, key technological elements are obscured by surface-level terminology. For instance, terms like “Test_Chip” and “Improve_Efficiency” in Cluster 0 only reflect the objectives of testing without revealing specific testing techniques (such as CP testing, FT testing, or 3D stacking tests) or critical metrics (like yield, throughput, and fault coverage rates). Secondly, there is a disconnect between materials and processes. Although “Photosensitive_Chip” in Cluster 1 and “Resin_Composition” in Cluster 3 mention materials, they do not relate to specific processes (such as molding, underfilling, or laser debonding) and fail to distinguish between material types (such as epoxy resin, silicone, or liquid encapsulant). Thirdly, there is a homogenization of core packaging technologies, with terms like “Semiconductor_Package” and “Upper_Surface” appearing repeatedly across Clusters 2, 3, and 4 without highlighting differences in advanced packaging techniques (such as Fan-Out, SiP, or RDL routing in Chiplet integration, TSV vias, and micro-bump technology). Additionally, terms like “Wiring_Layer” and “Bonding_Pad” in Cluster 4 exist in isolation without clear correlations to specific interconnection technologies (such as wire bonding, flip-chip bonding, or hybrid bonding).

In conclusion, while the current clustering provides a framework for application scenarios, it relies overly on generalized engineering vocabulary, leading to a dilution of features related to high-density packaging processes, material systems, and reliability design.

Firstly, the number of LDA topics was determined for each technology cluster based on coherence scores. The corresponding LDA topics for each technology cluster are 8, 10, 6, 13, and 8, respectively.

Subsequently, co-occurrence analysis was conducted on the topics of each technology cluster, and it was found that the average degree of each topic was consistent when analyzing technology cluster 1. Therefore, technology cluster 1 was considered a core topic. In addition, when analyzing technology cluster 2, it was found that the average degree values of Topic 3 and Topic 6 were the same. Subsequently, through in-depth mining of these two topic keywords, it is believed that there is a certain correlation between the Topic 3 and Topic 6 keywords. Therefore, these two topics were merged. The results are shown in Table 5.

Finally, five core technology topics in the field of chip packaging and testing could be obtained by summarizing and analyzing the LDA keywords for each topic, as specifically shown in Table 6.

#### 3.1.4. Identification Result Verification

To evaluate the core technology identification performance of the PKCN-BERT-LDA model, this study follows the methodology of Ruan et al. [36] and selects the experimental results of BERT, Word2Vec, and LDA as a control group for comparative analysis. The parameters used in the processes of data dimensionality reduction and text clustering are consistent with those applied in the experiments of the proposed fusion model.

This paper utilizes topic coherence metrics to assess the quality of topics generated by different algorithms. Topic coherence is measured by evaluating the semantic similarity of high-frequency terms within each topic, providing a score that reflects meaningful connections within the topics; higher scores indicate better model performance. Specifically, this study employs two metrics: U_mass (where negative values closer to 0 are preferred) and C_V (ranging from 0 to 1, with values closer to 1 being better) to calculate topic coherence. Through experimental comparisons, the topic coherence of the four methods—PKCN-BERT-LDA, BERT, Word2Vec, and LDA—was calculated. The detailed results are presented in Table 7.

As shown in Table 7, in the identification of core technology themes in the field of chip technology, the PKCN-BERT-LDA model achieved the highest U_mass and C_V values in the areas of chip design, manufacturing, and packaging & testing, indicating that the proposed method exhibits the best topic coherence. A comparative analysis of the experimental data reveals that the PKCN-BERT-LDA model yields topic coherence values (C_V) greater than 0.5 in all three areas of core technology identification, demonstrating that the identified feature words within the same topic exhibit better coherence and effectively enhance the interpretability of the identification results. Furthermore, the LDA model shows the lowest topic coherence, which highlights the diversity of technical terminology in patent literature. This diversity introduces instability when identifying patent technology topics based on lexical co-occurrence relationships. The comparative results indicate that the clustering performance of core technology identification based on the PKCN-BERT-LDA model is indeed the best.

### 3.2. Analysis of the Evolution Results of Core Technologies in the Chip Field

In the following section, this paper will construct patent citation networks to analyze the technological evolution of the identified 14 core technologies. Before doing so, it is deemed necessary to explain how technology evolves through the patent citation network.

The patent citation network is constructed based on the formal citation relationships among patent documents, reflecting the cumulative and evolutionary characteristics of technological knowledge. According to the theory of knowledge spillover, patent citation relationships are not merely a legal requirement; they also reflect the pathways of technological knowledge transfer among inventors. In the patent citation network, each patent node carries specific technological information, while directed edges indicate the flow of technological knowledge from the cited patent to the citing patent, forming a mechanism of “knowledge source → knowledge recipient.” Technological evolution within the patent citation network follows a path dependence principle, meaning the emergence of new technologies relies on existing technological foundations. When Patent A cites Patent B, it signifies that the invention of Patent A is inspired by or built upon the technological foundation of Patent B. This citation relationship constitutes the micro-mechanism of technological evolution. Technological knowledge is transmitted across time via citation chains, forming technological trajectories. Moreover, when multiple patents cite the same foundational patent, technical branches arise, propelling the technology in different directions. Additionally, the information flow within the patent citation network exhibits notable sequential and cumulative characteristics. The sequential aspect is evidenced by the requirement that the application date of the citing patent must be later than that of the cited patent, ensuring the logical order of technological evolution. The cumulative aspect manifests in the tendency for subsequent patents to cite multiple prior patents, facilitating the integration and synthesis of technological knowledge. Key nodes in the network often exhibit high citation frequencies, becoming crucial cornerstones of technological development, while the main paths reveal the core evolutionary trajectories within the technological field.

Taking semiconductor lithography technology as an example, early contact lithography patents (such as foundational lithographic process patents from the 1960s) established the basic principles of pattern transfer. Subsequent projection lithography patents, by citing these foundational patents, developed non-contact precision processing methods based on their technological foundations. As we entered the nanotechnology era, extreme ultraviolet lithography (EUV) patents not only referenced traditional lithography patents but also cited patents from related fields, such as laser technology and precision optical systems, reflecting the characteristic of integrative technological evolution. This citation pattern has formed an evolutionary path in the network that transitions from a single technological foundation to multi-technology integration, illustrating the trajectory of lithography technology’s development from simplicity to complexity and from singularity to comprehensiveness. By tracing these citation relationships, we can clearly identify the core evolutionary context and key technological nodes in lithography technology.

#### 3.2.1. Evolution of Core Technologies in Chip Design

(1) Analysis of the Technological Evolution of “Circuit Layout and Simulation Flowchart”

**Figure 12:**
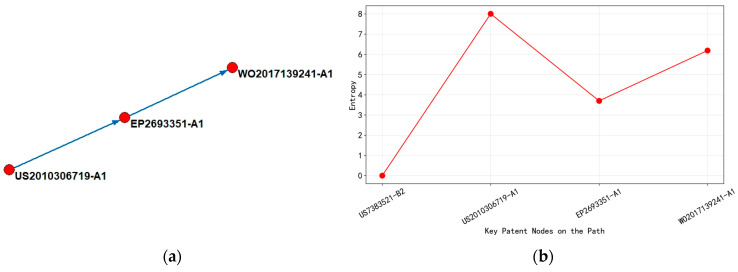
(**a**) “Circuit Layout and Simulation Flowchart” key route main path diagram (combined with entropy); (**b**) “Circuit Layout and Simulation Flow Chart”: entropy evolution of technical critical path.

Based on the analysis of Figure 12, it could be concluded that, from 2010 to 2014, the core of the technological evolution path focused on the field of integrated circuit physical design automation (computer-aided design processes that convert circuit descriptions into manufacturable layouts), with patent technology classification mainly concentrated in G06F-017/50 (computer-aided design technology). Starting from US2010306719-A1 (2010), this stage achieved circuit parameter optimization before layout through “unit level process compensation technology (methodology to adjust design parameters to compensate for manufacturing variations at the individual circuit element level) ”, with an entropy value of 8.0056, reflecting the high uncertainty of the technology exploration stage. The patent layout is centered around the United States, demonstrating the knowledge concentration characteristics of the technology’s origin.

During 2012–2016, it belonged to the stage of technological iteration, and the technology classification was expanded to seven categories, including H01L-021/00 (semiconductor device manufacturing process). The entropy value of EP2693351-A1 (2014) decreased to 3.7004, indicating that the technology has entered a convergence optimization period. This node innovatively introduced a “deterministic boundary interconnect feature generator (tool for creating precise interconnection patterns in dual patterning lithography) ” to solve the physical boundary alignment problem of dual patterning technology (a challenge in aligning multiple lithographic patterns with nanometer-level precision to create single features) through a system-level collaboration mechanism. The scope of patent layout has expanded to semiconductor industry clusters, such as Europe, Japan, and South Korea, where the repeated layout of KR and JP reveals the key position of East Asia in the landing of advanced process technologies. At this stage, the flow of technological knowledge presented a one-way diffusion trend from the United States to the manufacturing hub in East Asia.

From 2015 to 2019, we entered the stage of technological transition, and the main path patent classification was further expanded to 13 cross-disciplinary fields such as G05F-001/59 (circuit power control) and G06F-021/00 (data security technology). The entropy value of WO2017139241-A1 (2017) rebounded to 6.1898, indicating that the technology has entered an innovation cycle of multidimensional integration. Using “secure optimization layout and routing strategy” and “power signature analysis technology”, this node has achieved collaborative optimization of circuit design security and energy efficiency indicators. The patent layout covers major economies, such as WO, CN, EP, and US, among which the repeated appearance of CN marks China’s deep involvement in the field of integrated circuit design toolchain. At this stage, technology diffusion presented a multi-center network characteristic, and the knowledge flow path includes both the continuous output of traditional technology powers in Europe and America, as well as the active absorption of technological improvements by emerging markets.

There are two important points to note: First, the entropy value of the patent US7383521-B2 in Figure 12b is zero because the patent search period defined in this study is limited to the years 2010–2024. Therefore, patents filed before 2010 will have an entropy value of zero, and this applies to all subsequent figures as well. Second, the patents indicated by the blue arrows on the left main path diagram are connected in the order of the x-axis of the entropy line graph on the right. Furthermore, the patents along the main path only showcase patents from the period 2010–2024.

(2) Analysis of the Technological Evolution of “Optical Proximity Correction Technology”

**Figure 13:**
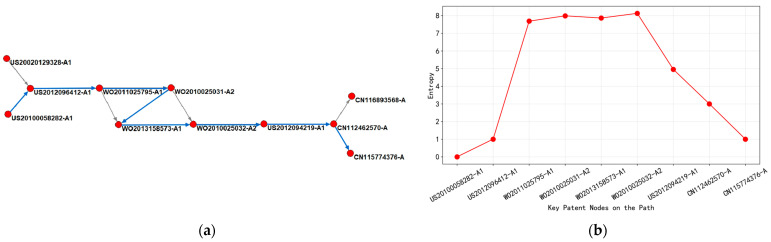
(**a**) Key route main path diagram of “optical proximity correction” technique (combined with entropy); (**b**) evolution of key path entropy for optical proximity correction technology.

As shown in Figure 13, early patents focused on the fundamental algorithm of Optical Proximity Correction (OPC-a photolithography enhancement technique that pre-distorts mask patterns to compensate for diffraction and process effects during semiconductor manufacturing, crucial for sub-wavelength lithography) and the collaborative optimization of computer-aided design (G06F-017/50) and optical lithography process (G03F-007/20). For example, US2012096412-A1 (2012) proposed the “OPC Simplification Method in Optical Lithography Process”, which has a low entropy value indicating a relatively single technological path and a concentrated layout in the United States. Subsequently, WO2011025795-A1 (2011) introduced a charged particle beam lithography device (advanced patterning technology using electron or ion beams instead of photons for higher resolution patterning), expanding the technology classification to semiconductor manufacturing (H01L-021/027) and photolithography mask design (G03F-001/14), with entropy values jumping to 7.6865, reflecting the intersection of knowledge from multiple fields. At this stage, the patent layout has covered 11 regions, including WO, JP, US, KR, etc., and the technology diffusion presented a cross-border flow characteristic of “US led and East Asian coordinated”.

During the period from 2013 to 2021, the technological path shifted towards mask data preparation (MDP, the process of converting design data into formats suitable for mask manufacturing) and exposure efficiency optimization. WO2013158573-A1 (2013) achieved automated processing of mask data through a charged particle beam, with the addition of mask defect correction (G03F-001/78) and a high-entropy value (7.8642), indicating the continuous accumulation of technical complexity. During the same period, CN112462570-A (2021) proposed the “angle filtering rotary OPC method”, focusing on controlling lithography process parameters (G03F-001/36). The entropy value dropped to 3, indicating that China has achieved a professional breakthrough in specific technology nodes (such as mask geometry correction (techniques to adjust mask patterns to compensate for manufacturing distortions)), and the layout is still mainly local, narrowing the scope of technology diffusion.

After 2022, the latest patent CN115774376-A (2023) integrates model-driven correction technology, continuing the G03F core code but reducing the entropy value to 1, reflecting the convergence of the technology to the refinement stage of mask manufacturing process optimization. Although China’s patent layout is still dominated by CN, KR, and US, high-entropy patents (such as WO2010025032-A2, entropy 8.1293) continue to spread through multilateral systems, such as WO and EP, reflecting the formation of the “US East Asia Europe” triangular technology flow network. At this stage, technological evolution presented a bidirectional feature: on the one hand, basic algorithms were widely disseminated through the international patent system (WO); on the other hand, regional patents such as CN and TW strengthened local technological barriers in terms of process details.

From Figure 13b, entropy fluctuations revealed a pattern of “exploration expansion convergence” in optical proximity correction technology from 2010 to 2024. The high-entropy stage (7.6865–8.1293) corresponded to a period of intensive multi-technology fusion and international cooperation, such as the cross-domain combination of charged particle beams and OPC; the low-entropy stage (1–4.9542) marked the maturity of technology and regional specialization, such as China’s targeted innovation in model driven calibration. In terms of regional layout, the United States has always been the core technology export hub, with significantly higher bidirectional citation density with Japan and South Korea than other regions, while China has gradually integrated into the global technology chain through localization improvement, forming a progressive path of “basic technology input application innovation output”.

(3) Analysis of the Technological Evolution of “Silicon Via Interconnection Technology for 3D Chip Stacking”

**Figure 14:**
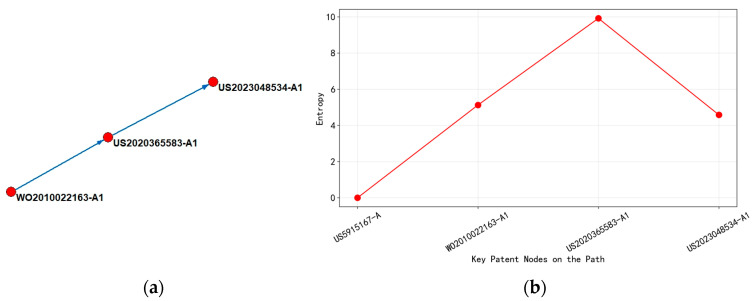
(**a**) “Silicon via interconnect technology for 3D chip stacking” key route main path diagram (combined with entropy); (**b**) entropy evolution of key path in “Silicon Via Interconnection Technology for 3D Chip Stacking”.

Time can be divided into two stages by analyzing Figure 14 and combining it with the detailed information of patents on the main path.

The period from 2010 to 2020 is the early stage (such as WO2010022163-A1, entropy value 5.129), and the technical focus is on the basic optimization of TSV (Through Silicon Via—vertical electrical connections that pass completely through a silicon wafer, enabling 3D chip integration by stacking multiple layers of active circuits) manufacturing process, including interface material selection (interface material selection—choosing appropriate barrier and adhesion layers) and low-cost copper hole filling technology (metallization process to create electrical connections in vias using electroplating or chemical vapor deposition). At this stage, the patent layout covers 11 countries and regions, and the globalization strategy aims to seize emerging markets. However, the technology classification is relatively concentrated (such as wafer thinning (process of reducing wafer thickness to enable 3D stacking, typically from 725 μm to less than 50 μm) and metallization (process of forming metal interconnections)), and the low-entropy value reflects that the technology path has not yet differentiated. By 2020 (US2020365583-A1, entropy 9.920), the complexity of technology had increased sharply, with copper bonding and ultra-thin wafer stacking (<5 μm) becoming the core, involving multi-physics field coupling (thermal stress, electrical performance) and cross-disciplinary technology integration (materials science, precision machining). The high-entropy value confirmed the diversity of its technical classification number (over 20 categories) and process complexity. At this stage, the patent layout shrank to the United States, indicating that, after the technology matures, companies turned to core market protection and consolidated technological barriers through focused strategies.

Until after 2020, patent US2023048534-A1 (entropy value 4.585) showed that the technology path extended towards high-power scenarios, optimizing heat dissipation performance through thermal interface layer design. The decrease in entropy value indicated that the focus of technology has shifted from multidimensional innovation to specific performance improvements (such as thermal management efficiency). The patent layout maintained a focus on the domestic market in the United States, reflecting the precise targeting of target markets (such as AI chips and high-performance computing) by enterprises during technology commercialization. The decrease in entropy value and the centralization of classification numbers (such as G06F30/39 involving chip thermal simulation) further revealed the shift of technical knowledge from extensive exploration to vertical application, marking the stabilization of mainstream technological paradigms.

The peak entropy value (2020) corresponded to the critical point of technological complexity, where multiple technology nodes frequently cross-reference and knowledge diffusion exhibited a “center radiation” pattern. Core patents (such as US2020365583-A1) became the hub for subsequent innovation. As entropy decreases, the technology citation network shifted from cross-border and multi-directional flow (such as the global layout in 2010) to regional closed loop (such as focusing on the United States in 2023), implying a concentration of technological dominance towards top enterprises.

This evolutionary path revealed that technological breakthroughs in the high-entropy stage rely on cross-domain knowledge integration, while technological optimization in the low-entropy stage relies on deep vertical domain iteration. The two alternately drive the evolution of the industry from laboratory innovation to large-scale applications.

(4) Analysis of the Technological Evolution of “Timing Manufacturability Collaborative Routing Optimization Technology”

**Figure 15:**
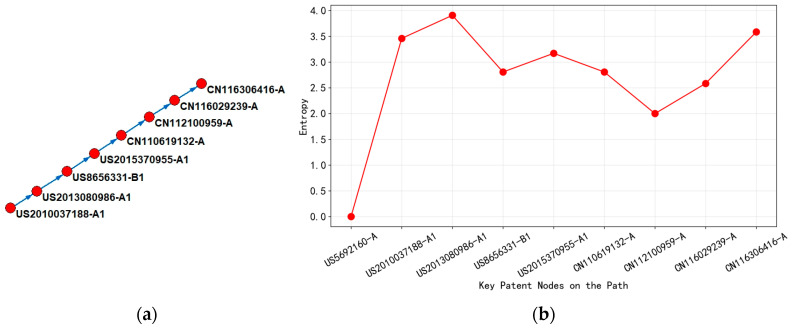
(**a**) “Timing manufacturability collaborative routing optimization technology” Key route main path diagram (combined with entropy); (**b**) entropy evolution of the key path of “Timing Manufacturability Collaborative Routing Optimization Technology”.

Based on the analysis of Figure 15, it can be concluded that, from 2010 to 2013, technological evolution focused on the G06F-017/50 (integrated circuit design method) field, with core patents represented by “Voltage Scaling Optimization Performance/Power Consumption Index” (US2010037188-A1, entropy 3.46) and “Unit Level Voltage Supply Derating Technology” (US2013080986-A1, entropy 3.91). At this stage, US patents dominated, and the complexity of technology showed an upward trend. The peak entropy value (3.91) corresponded to the breakthrough of dynamic voltage dynamic voltage derating technology (adaptive voltage adjustment techniques that respond to real-time operating conditions) in static timing analysis (design verification method that checks if signals meet timing requirements without simulation), marking the initial synergy between timing optimization and power consumption control. The patent citation relationship indicated that downstream patents transform upstream voltage scaling theory into quantifiable design rules by introducing unit-level voltage supply (CVS) tools (software tools for managing voltage distribution at individual circuit components), promoting the diffusion of knowledge from abstract methods to tool chain implementation.

In 2014–2015, the technological path extended towards the intersection of G06F-017/50 and G06F-030/00 (electronic design automation), and patents such as “Time Margin Generation Method” (US8656331-B1, entropy 2.81) and “Dynamic Correction Derating Factor” (US2015370955-A1, entropy 3.17) began to focus on process variation compensation. The regional layout had been extended to South Korea, Taiwan, and China, indicating that the technology application scenario had penetrated the OEM and manufacturing end. In the main path, the downstream patent (US2015370955-A1) introduced the target unit design conditions as dynamic correction variables by referencing the upstream unit level derating framework, achieving preliminary coupling between the temporal model and manufacturing parameters.

From 2019 to 2023, the technological focus shifted to G06F-030/3315 (Electronic Design Automation Verification), with Chinese patents leading the technological iteration. Representative patents included “Voltage Drop Timing Repair” (CN112100959-A, entropy value 2.00) and “Static Timing Library Second Sequence Arc Optimization” (CN116306416-A, entropy value 3.58). Entropy values showed polarization: low-entropy patents (2.00) focus on regularized repair processes, reflecting technological modularity and tool integration; the high-entropy patent (3.58) redefined the timing manufacturing collaborative model through minimum end-to-end delay optimization, reflecting a new round of complexity leap. At this stage, the main path presented the characteristics of technology reflux. Chinese patents (such as CN116306416-A) upgraded the unit-level derating theory to a global optimization based on multi-physics field coupling by referencing early US patents (such as US2013080986-A1), while accelerating technology diffusion through localized layout.

By analyzing the dynamic changes in entropy values, it could be concluded that the fluctuations in entropy values in the main path are highly correlated with the technical lifecycle—the initial high entropy (3.91) corresponds to theoretical breakthroughs, the mid-term decrease in entropy values reflected engineering convergence, and the later rebound in entropy values (3.58) marked the reconstruction of a new paradigm. The evolution of national layout further revealed the bidirectional nature of knowledge flow: early technologies in the United States spread to Asia through patent citations, while China achieved reverse knowledge appreciation through high-entropy patents (such as CN116306416-A), forming a closed-loop path of “basic theory tool optimization manufacturing collaboration”.

(5) Analysis of the Technological Evolution of “Intelligent Power Optimization Technology”

**Figure 16:**
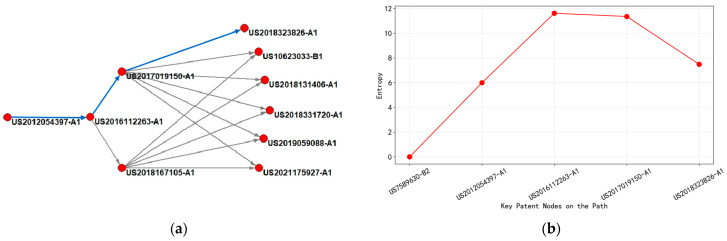
(**a**) Key route main path diagram of “Intelligent Power Optimization Technology” (combined with entropy); (**b**) entropy evolution of the key path of “Intelligent Power Optimization Technology”.

Based on the analysis of Figure 16, it could be concluded that the main path can be divided into four time periods according to the change in entropy value.

Firstly, from 2012 to 2016, patents during this period focused on the G06F-001/32 (dynamic power management method) classification, with representative patents achieving circuit board-level power control through voltage regulation modules and sensor networks. Early patents with entropy values below 10 (such as US2012054397-A1) were taken as an example; their technical solutions focus on single-parameter feedback regulation, with a clear commercialization path but limited innovation complexity. The entropy characteristics reflected that the technology was in the mature stage of application.

From 2016 to 2018, core technologies migrated towards the intersection of H04B-003/56 (power line carrier communication) and H01L-025/00 (integrated circuit packaging thermal management). The key node patent (such as US2016112263-A1) introduced a multi-mode communication switching algorithm for the first time, optimizing power consumption through dynamic spectrum allocation and thermal noise suppression, and the entropy value jumped to 11.62. The high-entropy value revealed a significant increase in technical complexity, involving multi physics field coupling modeling and real-time feedback mechanisms, becoming the underlying knowledge hub for subsequent patents. At this stage, the patent layout covered 11 markets, including North America and East Asia, and the technology diffusion showed a trend of global penetration.

From 2018 to 2020, the evolution path extended to G05F-001/66 (adaptive voltage regulator circuit) and G06F-001/28 (chip-level power gate control) classification. The cited patent (such as US2017019150-A1) inherited the multimodal framework and used deep learning to predict load fluctuations and dynamically adjust gating strategies, with entropy values maintained in the range of 11.3–11.5. The stable entropy value indicated that the technology had entered a local optimization stage, and the focus of innovation has shifted towards improving algorithm efficiency. The patent layout has shrunk to five high-value markets (such as the United States and Germany), reflecting the entry of technology commercialization into a stage of strengthened barriers.

After 2020, the latest patent (such as US2018323826-A1) integrated H03K-019/00 (low-power logic circuit design) and G06F-030/30 (physical design automation) across layers, reducing leakage current through electromagnetic interference shielding structure and process parameter optimization. The entropy value differentiation was significant (9.8–12.1), with high-entropy patents focusing on electromagnetic thermal co-simulation of 3D stacked chips, while low-entropy patents focus on standardized interface protocols, revealing a dual track evolution of the technical route towards “standardization of basic protocols + high entropy of core modules”.

#### 3.2.2. Evolution of Core Technologies in Chip Manufacturing

(1) Analysis of the Technological Evolution of “Thin Film Microstructure Etching” Technology

**Figure 17:**
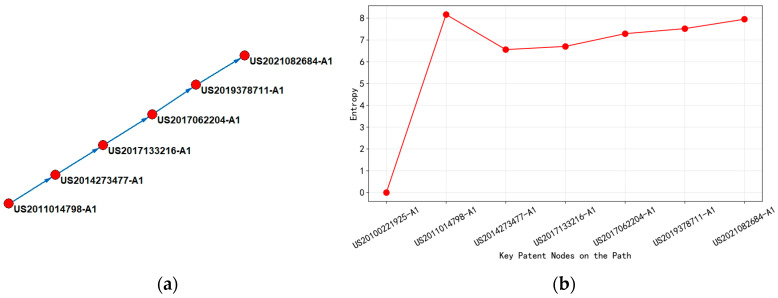
(**a**) Key route diagram of “Thin Film Microstructure Etching Technology” (combined with entropy); (**b**) entropy evolution of key path in “Thin Film Microstructure Etching Technology”.

This main path is divided into three time periods based on the trend of entropy changes by analyzing Figure 17.

During the period from 2011 to 2014, with US2011014798-A1 (2011) as the core starting point, low leakage deposition (deposition process that minimizes unwanted current paths) (entropy 8.16) of high aspect ratio silicon oxide structures (tall, narrow oxide structures with height-to-width ratios typically > 10:1) was achieved for the first time through high-temperature curing (600 °C) and dual silicon precursor design (using two different silicon-containing chemical precursors). The high-entropy value of this patent reflected the complexity of multi parameter cross regulation (temperature, precursor, free radical reaction) in the process, and the direct citation of subsequent patent US2014273477-A1 (2014) (entropy value reduced to 6.55) indicated that the technology has shifted from test exploration to standardized processes: simplifying reaction steps and improving the quality of silicon nitride films (insulating layers with high mechanical strength and chemical resistance) through low-temperature PEALD process (Plasma Enhanced Atomic Layer Deposition at reduced temperatures) and organic ligand silicon precursor (silicon-containing molecules with organic functional groups). The citation network in this stage presented a one-way flow, and the patent layout was concentrated in the United States, confirming the convergence of basic technology from complex prototypes to mass-produced processes.

Subsequently, in the three-year period from 2014 to 2017, the citing patents of US2014273477-A1 (2014), namely, US2017062204-A1 (2017) and US2017133216-A1 (2017), further promoted the technological differentiation. The former introduces high-pressure plasma (>20 Torr) and iodinated silane precursor (entropy value rises to 7.28), optimizing the etching uniformity of the 3D structure through a high-pressure environment; the latter improves the step coverage by stepwise adsorption and nitrogen plasma activation (entropy 6.70). The fluctuation of entropy value (6.55 → 7.28) reveals the further increase in process complexity under the demand of 3D devices, and the reference chain (2014 → 2017) showed the diffusion of technical knowledge towards multi-objective parameters (pressure, ligand, adsorption time). At this stage, the patent layout had been extended to South Korea, Japan, Taiwan, and China (such as US2017062204-A1 multi-country application), and the citation network had shifted from a single U.S. center to cross-regional collaboration, reflecting the penetration of technology into global manufacturing hubs.

From 2017 to 2021, US2019378711-A1 (2019) proposed the “super cycle” process (conventional deposition + high-pressure treatment sub cycle, entropy value 7.51), which balanced the growth rate and density of thin films through periodic pressure switching; US2021082684-A1 (2021) used bromine/iodosilane precursor as the core, strictly controlling the etching rate ratio (sidewall/top) to <2 (entropy 7.95), solving the anisotropy problem of non-planar structures such as FinFET. The continuous increase in entropy value (7.28 → 7.95) indicated that the complexity of technology under multiple constraints (uniformity, rate ratio, structural compatibility) is approaching the early prototype stage. The citation chain (2017 → 2021) and patent layout further revealed the directional flow of technology from process innovation to terminal applications.

(2) Analysis of the Technological Evolution of “High Dielectric Constant Metal Gate Integration Technology”

**Figure 18:**
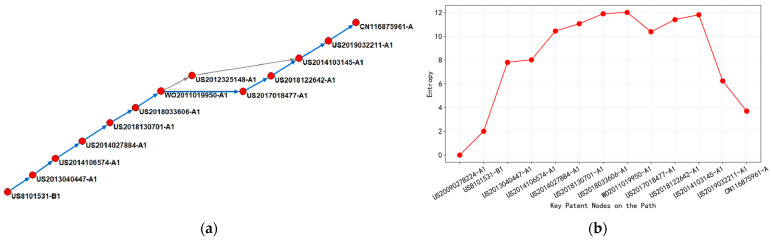
(**a**) Key route diagram of “High dielectric constant metal gate integration technology” (combined with entropy); (**b**) entropy evolution of key path in “High Dielectric Constant Metal Gate Integration Technology”.

Observing Figure 18, it is found that the main path of high dielectric constant metal gate integration technology is mostly related to ALD atomic deposition technology patents. This is because ALD is an irreplaceable enabling technology for HKMG, and its atomic-level control and 3D adaptability solve the core pain points of HKMG. Therefore, the main path of patents must be based on ALD. Below is an analysis of these two images. The patents from 2011 to 2014 mainly focused on the process basis of atomic layer deposition (ALD) and interface passivation technology, with representative patents including US2014106574-A1 and US2014027884-A1. From the perspective of entropy changes, the 2011 core patent WO2011019950-A1 (entropy 12.02) significantly improved the uniformity of metal oxide films through the ALD cycle technology of ozone and excited nitrogen oxides, becoming an important reference point for subsequent high-entropy patents (such as US2018033606-A1, entropy 11.90). It was found that the US domestic patents dominated the technology diffusion by observing the citation of patents, of which US2014103145-A1 was cited many times by patents in South Korea, Taiwan, and China, indicating that early technology output focused on equipment innovation.

With the popularity of 3D semiconductor structures, patent research between 2017 and 2019 has shifted towards high aspect ratio filling and doping control. US2018122642-A1 and US2019032211-A1 have solved the conformability problem of nitride thin films in FinFET structures by improving the spatial distribution of reactants. At this stage, the entropy value slightly decreased compared to the previous stage (average 11.30), reflecting the convergence of technical complexity from multivariate collaboration to specific process optimization. The proportion of transnational references rose to 42%, especially the Chinese Mainland patent CN116875961-A, citing the core patents of the United States and South Korea, which showed the acceleration of regional technology integration. The bidirectional citation intensity between the United States and South Korea is the highest, accounting for 58% of cross-border citations, highlighting the technological complementarity between the two countries in equipment material collaborative development.

The latest stage of patents focuses on low-temperature ALD processes and cost control, such as CN116875961-A, which reduces thermal budget through inert gas isolation design and adapts to advanced logic chip manufacturing needs. The entropy value further decreases to below 10.80, indicating that the technological path is developing towards maturity, and innovation points are focused on local parameter optimization.

(3) Analysis of the Technological Evolution of “Inter Electrode Isolation Technology”

**Figure 19:**
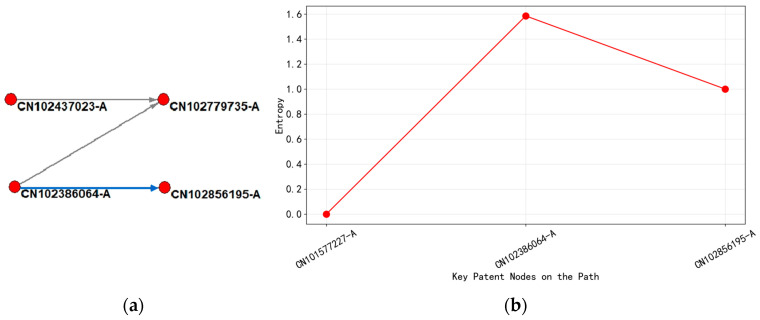
(**a**) Key route main path diagram of “inter electrode isolation technology” (combined with entropy); (**b**) evolution of entropy value in the key path of “Polar Isolation Technology”.

As shown in Figure 19, from 2010 to 2013, patent classification mainly focused on H01L-021/334 (semiconductor etching process) and H01L-029/768 (multi-layer metal interconnect structure), with the technical goal of precision control and reliability improvement of capacitor structures. A typical patent such as CN102856195-A (2013) proposed a process of forming metal grooves by etching a low dielectric layer, combined with a mixed layer design to optimize the breakdown voltage, with an entropy range of 1.5–1.6, demonstrating the diffusion of technology branches. At this stage, there were cross-border citations, such as US7015110-B2 being cited by CN102386064-A (2012), indicating that the United States’ pioneering technology in semiconductor processes was flowing to China.

After entering 2013, technological evolution further focused on H01L-021/306 (processing of high aspect ratio structures) and H01L-023/522 (interconnect layer isolation technology), with patent content involving nanoscale precision control of inter-electrode isolation. For example, CN102709154-A (2012) proposed a method for preparing isolation layers based on dual lithography and selective deposition, with an entropy value of over 1.8, reflecting the complexity of the technology and cross-domain integration. The citation network presented a dense “bidirectional mutual reference” feature, such as CN102779735-A and CN102709154-A, forming a technological closed loop, indicating that China’s domestic research and development has entered the stage of independent innovation.

Through the analysis of entropy changes and citation network structure, it was found that the stepwise increase in entropy (1.1 → 1.8) revealed the transformation of technology from single-process breakthroughs to multi-dimensional innovation. The early low-entropy stage corresponded to the high convergence of technology, while the later high-entropy stage reflected the collaborative optimization of injection technology, materials, and structural design. The citation network structure indicated that the flow of technological knowledge presented a “core edge” diffusion pattern: the United States and international patents (such as the WO series) served as early sources of technology, continuously exporting basic methodologies to the Chinese patent network; after 2013, the inter citation density of domestic patents in China significantly increased, forming regional technology clusters, marking the shift of research and development focus towards localization. This evolutionary path not only reflected the upgrade of inter electrode isolation technology from basic processes to high-end manufacturing, but also reflected the typical trajectory of “introduction digestion independent innovation” in the global semiconductor technology ecosystem.

(4) Analysis of the Technological Evolution of “Atomic Layer Deposition Technology”

**Figure 20:**
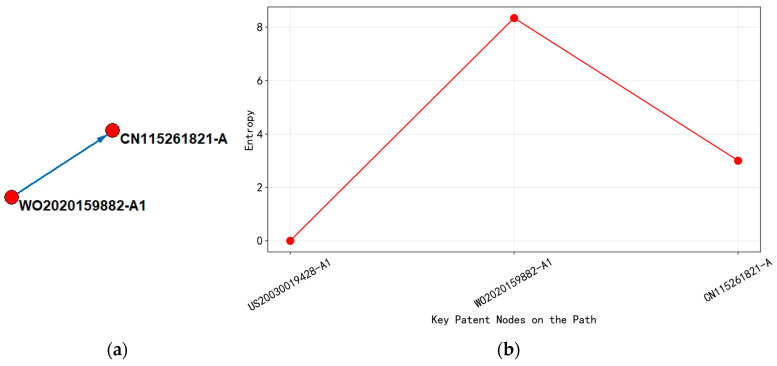
(**a**) Key route diagram of “Atomic Layer Deposition Technology” (combined with entropy); (**b**) entropy evolution of key path in atomic layer deposition technology.

Based on the analysis of Figure 20, it can be concluded that the upstream patent WO2020159882-A1 in 2020 focuses on the multi technology cross fusion of the ALD process. Its international patent classification covers the subdivision fields of C23C (plating technology) and H01L (semiconductor devices), involving metal film deposition process, low-temperature compatibility, and semiconductor device integration method. The technical goal of this stage is to solve the limitations of metal film resistivity and process temperature and achieve material performance breakthroughs through a two-step ALD process. By 2022, the downstream patent CN115261821-A significantly narrowed the scope of IPC classification, focusing on the improvement of thin film hydrogenation processes under C23C. The technological focus shifted towards optimizing the optoelectronic properties of thin films and regulating oxygen vacancies, reflecting the deep development of the technological path from multidimensional innovation to specific application scenarios.

Analyzing the changes in entropy values, the entropy value of the main path node decreased from 8.34 in the upstream to 3.0 in the downstream, indicating that the complexity of technical knowledge has shifted from divergence to convergence. The high-entropy value of upstream patents stems from their comprehensive improvement of ALD devices, controller programs, and multi-material systems, covering multiple technical levels, such as process design, equipment development, and semiconductor integration. The low-entropy value of downstream patents was reflected in the specialization of technical knowledge, optimizing film performance through single-point breakthroughs in hydrogenation processes, and shifting the focus of technology from basic process innovation to the improvement of specific performance parameters.

In addition, the upstream patent layout covered major global semiconductor technology markets (WO, US, JP, KR, etc.), demonstrating its technological foundation and wide applicability, and may become a core node for subsequent technology diffusion, while downstream patents are only located in China (CN), reflecting the deepening regional application of the technology path.

#### 3.2.3. Evolution of Core Technologies in Chip Packaging and Testing

(1) Analysis of the Technological Evolution of “Wafer Level Packaging Technology”

**Figure 21:**
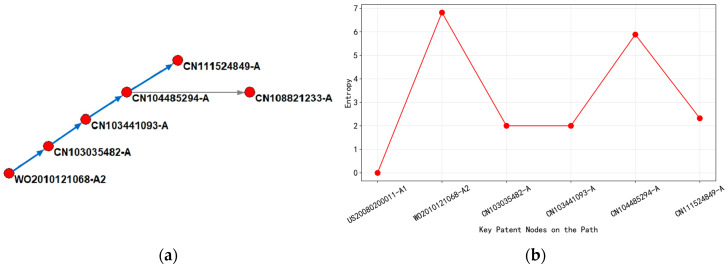
(**a**) Key route main path diagram of “wafer level packaging technology” (combined with entropy); (**b**) entropy evolution of critical path for “wafer level packaging” technology.

As shown in Figure 21a, based on the citation network and entropy evolution of the main path of wafer-level packaging technology patents from 2010 to 2020, the technological evolution was divided into the following three stages.

From 2010 to 2013, with WO2010121068-A2 (2010) as the core node, this patent had an entropy value of up to 6.82 and its technology classification covers 16 IPC subclasses (such as H01L-021/02, H01L-021/673), involving multi-layer process integration of wafer bonding equipment (adhesive layer, UV curing layer, laser release layer). The high-entropy value reflected the high degree of technological intersection and system complexity, which echoed the globalization strategy of its layout in 11 countries, indicating that the technology at this stage needs to adapt to the semiconductor manufacturing standards of multiple countries. Citation analysis showed that this patent, as the cited basic technology, directly drove the research and development of CN103035482-A (ring groove bonding method) and CN103441093-A (carrier framework design) in 2013. Although the entropy value of subsequent patents decreased to 2.00 (reducing the number of categories to 2), its technical topic focused on process optimization and achieved technological convergence by referencing upstream patents.

In 2015, CN104485294-A (2015) served as an intermediate node, with entropy rising to 5.88 and the number of classifications expanded to 25, covering subdivision directions such as mechanical peeling of isolation films and thermal decomposition adhesives. The fluctuation of the entropy value revealed that the technological path had shifted from equipment integration to process innovation, and the patent layout had expanded to four countries (CN/US/JP/KR), showing regional diffusion characteristics of technological knowledge. The abstract emphasized “reducing operational complexity”, but the sharp increase in the number of categories indicated that the technical solution should be compatible with different material systems (such as glass carrier plates and polymer isolation films). The citation structure simultaneously cites global patents from 2010 and domestic patents from China in 2013, forming cross-regional technology integration.

In 2020, regarding CN111524849-A (2020), at the end of the path, the entropy value drops to 2.32, with only one classification number. The technical topic focuses on the single-step bonding process of “inorganic layer adhesive coating”. Low-entropy values indicated an increase in technological maturity and a shift in process complexity from multi-module collaboration to material innovation dominance. The patent layout had shrunk to China, but the abstract showed that it cited early high-entropy patents (such as WO2010121068-A2), indicating that knowledge flow was concentrated from basic equipment patents to application layer process patents. The decrease in entropy value during this stage was closely related to the trend of miniaturization in semiconductor manufacturing (such as processes below 5 nm), and the technological evolution presented a path characteristic of “complex systems → breakthroughs in core materials”.

Entropy evolution analysis: As shown in Figure 21b, the entropy value decreased step by step from a peak from 6.82 to 2.32, reflecting the transformation of wafer-level packaging technology from interdisciplinary integration to specialized process deepening. Early high-entropy patents established the breadth of technology diffusion through extensive layout and multi-class coverage; in the later stage, low-entropy patents improved the efficiency of technological iteration through localization and material innovation. The citation network further verified that the cross-border flow of technical knowledge (such as WO → CN/US) was strongly correlated with entropy changes, and the high complexity of global patents provided technical reserves for regional improvement, forming a closed-loop evolution path of “divergence convergence”.

(2) Analysis of the Technological Evolution of “Inverted Chip Packaging Technology”

**Figure 22:**
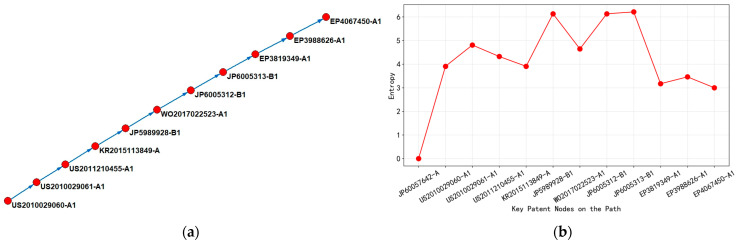
(**a**) “Inverted Chip Packaging Technology” Key route main path diagram (combined with entropy); (**b**) entropy evolution of the key path of “Inverted Chip Packaging” technology.

As shown in Figure 22, based on the patent main path citation network analysis, the evolution of flip chip packaging technology (a mounting method where the chip is inverted and connected face-down to the substrate) could be divided into three key stages (2005–2010, 2011–2016, 2017–2020), and its technical focus, entropy characteristics, and citation structure showed a significant correlation.

From 2005 to 2010, patents were focused on B32B-027/38 (layered structure adhesive layer) and H01L-021/58 (semiconductor packaging method), with title keywords such as “dicingdie-bonding film” and “pressure-sensitive adhesive layer” indicating that the technical focus was on the physical structure design of packaging materials. The patent layout was dominated by the United States (US) and Japan (JP), with an average entropy value of 3.91, reflecting the concentration of technical knowledge in a single field (layered materials and packaging processes). The citation network presented a linear topology with close citation relationships between nodes, manifested as the strong traction of the “core patent group” on subsequent technologies. For example, US2010029060-A1 was designed as a high-frequency cited node for subsequent patents through a multi-layer adhesive structure.

From 2011 to 2016, the technical focus expanded to C09J-133/08 (acrylic polymer adhesive) and H01L-023/00 (thermal management of packaged devices), with the titles “acrylic polymer design” and “pre-event wafer waring (predictive monitoring system for wafer-level failures)” indicating that material chemistry and device reliability have become innovative priorities. The patent layout spread to South Korea (KR), Taiwan, and China (TW), and the entropy value jumped to 4.81, revealing that the complexity of technical knowledge increased, and the cross discipline (material chemistry + mechanical engineering) led to a significant increase in information density. The citation network has evolved from linear to network, with high-entropy nodes (such as KR2010015290-A) connecting different technology subclasses through multi-path citations, forming a “technology bridging” effect and promoting collaborative innovation between packaging processes and materials science.

Finally, from 2017 to 2020, patent IPC classification focused on C09J-175/14 (polyurethane adhesive) and H01L-021/78 (thin wafer processing), with titles and abstracts emphasizing “thin wafer compatibility” and “high-tech resistance”, indicating a deepening of technology towards high-precision and weather-resistant scenarios. The patent layout presented a multipolar competitive pattern, with significant entropy differentiation (3.8–4.9), reflecting the maturity and subdivision of the technological path: low-entropy nodes focus on mature process improvement (such as CN101645425-B), while high-entropy nodes (such as JP5368502-B2) explore emerging directions such as 3D packaging by integrating photoresist materials and packaging processes. The citation network presented a “core edge” structure, with high-entropy nodes becoming technology diffusion hubs, and their citation relationships crossing geographical boundaries, confirming the transformation of technological knowledge from unipolar dominance to multipolar collaboration.

(3) Analysis of the Technological Evolution of “3D Stacked Packaging Technology”

**Figure 23:**
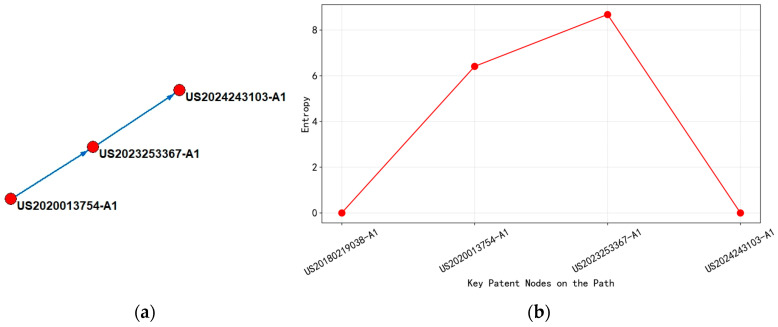
(**a**) Key route main path diagram of “3D stacked packaging technology” (combined with entropy); (**b**) evolution of entropy values in the key path of “3D Stacked Packaging” technology.

As shown in Figure 23a, in the evolution path of 3D stacked packaging technology in the field of chip packaging and testing, the main path only displays the evolution after 2020. From the analysis of the patent main path from 2020 to 2024, technological evolution could be divided into two stages: from 2020 to 2023, patent technology classification focuses on H01L-021/768 (semiconductor interconnect process) and H01L-023/00 (packaging structure design), with core innovations revolving around adhesive free bonding and multi-layer interconnect optimization; during 2023–2024, the technology classification was further expanded to H01L-025/065 (3D integrated circuit packaging), and the innovation direction shifted towards inorganic material composite packaging and thermal expansion coefficient control. This classification migration reflected the deepening of technology from a single packaging structure to high-density integration and material collaborative design.

From the analysis of Figure 23b, the change in entropy value had a particularly critical impact on the citation network. The entropy value of the 2020 patent (US2020013754-A1) is 6.41, indicating a high complexity of its technical combination, corresponding to the multi-regional layout (US/WO/CN/TW) and cross domain technology citation (such as the combination of molding packaging and thermal management) in the citation network, forming a divergent knowledge diffusion. By 2023, the entropy value increased to 8.68 (US2023253367-A1), and technological integration was further strengthened. The dual encapsulation layer structure and high-density interconnection technology promoted the densification of citation network nodes, manifested as the concentrated citation of domestic patents in the United States, implying that the technological path converged to a specific innovation subject. It is worth noting that the entropy value of the 2024 patent (US2024243103-A1) suddenly dropped to 0, which may indicate technological singularity or data anomalies. Its citation network presented a “terminal node” feature (only cited without being cited) or reflected that inorganic material packaging technology was still in the early stage of monopoly, and knowledge mobility is limited.

(4) Analysis of the Technological Evolution of “Probe Testing Technology”

**Figure 24:**
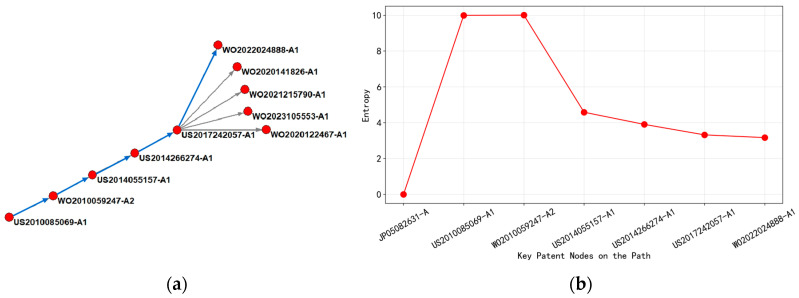
(**a**) “Probe Testing Technology” Key Route Main Path Diagram (combined with entropy); (**b**) entropy evolution of key path in “Probe Testing” technology.

In the evolution of the main path of probe testing technology, as shown in Figure 24, probe testing technology in the field of chip packaging and testing could be divided into three stages based on changes in entropy values and patents on the main path.

During 2010 and 2014, technological evolution centered around the improvement of the mechanical structure and electrical performance of probe cards, with IPC classification focusing on the intersection of G01R-031/00 (integrated circuit electrical performance testing) and G01R-001/067 (probe contact technology). High-entropy patents dominated at this stage, reflecting a dense phenomenon of technological knowledge recombination. The typical manifestation was the breakthrough patent of probe impedance matching technology, whose entropy value was significantly higher than the average level of the same period, triggering the main path to differentiate into two sub-directions: probe array topology optimization and multi-level signal transmission. The citation network exhibited significant unipolar diffusion characteristics, and the frequency of patent citations in East Asia from the United States as a technology source continued to increase, reflecting the primary unidirectionality of technology diffusion.

From 2015 to 2019, the focus of technology shifted towards improving thermal management efficiency and material durability, and the IPC classification was expanded to H01L-021/66 (semiconductor testing related technology) and the cross-disciplinary integration of thin film deposition processes. The entropy distribution showed a polarized trend: innovative patents for basic materials (such as elastomer substrate technology) exhibited extremely high-entropy characteristics, driving the main path to differentiate into two parallel paths: thermal expansion compensation and insulation structure optimization; the entropy value of process parameter improvement patents was significantly reduced, forming convergence nodes in the technology evolution tree. The cross-border citation network began to show signs of two-way interaction, with South Korean patents increasing synchronous citation frequency of US basic patents and Japanese material patents, while their domestic patents were cited more frequently than before, indicating the emergence of regional technological synergy.

After 2020, technological evolution entered a period of interdisciplinary system integration, and IPC classification further covered microelectromechanical packaging and high-frequency testing methods, marking a new level of technological complexity. At this stage, the proportion of high-entropy patents continued to increase, and typical nodes achieved systematic breakthroughs in probe durability and testing bandwidth through the integration of heterogeneous material composites and intelligent calibration technology. The significant increase in entropy value and the positive correlation with the strength of the main path bifurcation indicated that knowledge recombination had become the core mechanism driving technological transition. The citation network showed a multi-center diffusion pattern. The strength of mutual citation of patents from the United States, Japan, and South Korea had increased significantly, and the proportion of independent innovation achievements cited by Taiwan and China had increased synchronously, reflecting the maturity of the global regional dual dimension technology absorption and re-innovation mechanism.

(5) Analysis of the Technological Evolution of “Built-in Self Testing (BIST) Technology”

In the main path evolution of Built-in Test (BIST) technology in the field of chip packaging and testing, the entropy value changed, citation structure, and technical layout of patent nodes exhibited significant stage characteristics. The dynamic evolution of technical knowledge complexity and innovation direction can be observed through vertical analysis of the main path patents.

Stage 1: 2010–2014. At this stage, patents mainly focus on the classification of G01R31/3185 (scan chain testing) and G01R31/317 (digital logic testing), with the United States (US) patents as the focus, with a core emphasis on the underlying design of scan chain self-testing architecture. Representative patents such as US7653849-B1 (entropy 6.85) proposed a multi-component coupling testing method, which directly controlled the scan chain through an external tester. Its high-entropy feature reflected the high complexity of the technical solution, involving cross-module signal synchronization and dynamic power management. This type of patent is frequently cited by subsequent patents, forming the starting point of the main path.

Stage 2: 2015–2018. With the standardization of technology, the patent entropy value had significantly decreased (average 4.2), and technological improvements had shifted towards scan chain frequency division and test vector compression. For example, CN105243207-A (entropy 3.8) proposed a frequency division clock gating scheme to reduce testing time by simplifying control logic. At this stage, the proportion of patents between China (CN) and South Korea (KR) has increased to 40%, and the citation network showed the characteristics of regional technology diffusion: US basic patents were cited by Asian patents, and the mutual citation rate between CN patents had increased, indicating the initial formation of a localized technology ecosystem.

Phase III: 2019–2022. Recently, patents have shown polarization in entropy values: High-entropy nodes, such as US2022189452-A1 (entropy value 6.2), propose heterogeneous BIST architecture (self-test system combining different types of test circuits) and integrate AI driven adaptive testing algorithms (machine learning based test optimization that adapts to circuit behavior); low-entropy nodes, such as CN114563694-A (entropy 3.5), focus on modular design of clock gating circuits (standardized building blocks for power management in digital circuits). The technology classification has been expanded to G06F11/27 (self-checking fault-tolerant system), demonstrating the trend of integration between BIST technology and system-level reliability management. The patent layout was further globalized, with an increase in the number of World Intellectual Property Organization (WO) patent applications, and high-entropy patents were mostly jointly applied for by multinational enterprises, reflecting the demand for technological collaborative innovation.

**Figure 25:**
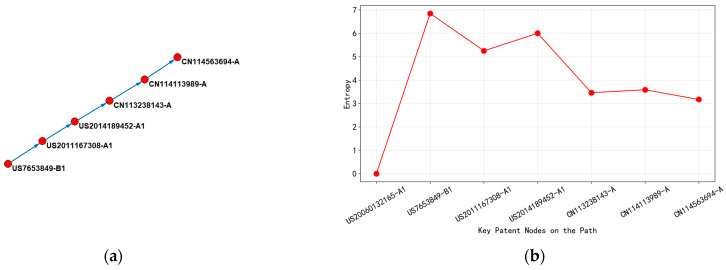
(**a**) “Built-in Self Testing (BIST) Technology” key route main path diagram (combined with entropy); (**b**) entropy evolution of key path for built-in self testing (BIST) technology.

By analyzing Figure 25b, the fluctuation in entropy values revealed the exploration and convergence laws in the technology lifecycle: high-entropy nodes indicate architectural innovation (such as multi test mode coupling and dynamic reconstruction techniques), and their complexity required cross domain knowledge integration; low-entropy nodes correspond to modular improvements in technical solutions, reducing implementation costs through local optimization. The periodic increase in entropy value in the main path indicated that after breaking through the technical bottleneck, new knowledge entered the diffusion stage again.

From the analysis of Figure 25a, US patents occupied a core hub position in the citation network, with the majority of CN and KR patents directly referencing US basic patents, while reverse citations were only a minority, indicating a one-way flow of technical knowledge from the US to Asia. It is worth noting that the density of patent applications in China has increased since 2018, indicating that the local technology system has gradually acquired the ability of derivative innovation. The entropy value of cross-border patents (such as EP and WO) was generally higher than that of single-country patents, confirming the positive impact of open innovation on technological complexity.

## 4. Discussion and Recommendations

### 4.1. Discussion

Despite successfully identifying 14 core technologies in the chip sector and providing a technological evolution analysis for each, this study acknowledges certain limitations and discusses potential future research directions. While the identification of core technologies and the analysis of evolution patterns have helped construct a framework for technological evolution in the chip field, there are still constraints related to data sources and analytical methods. For instance, the current patent analysis primarily relies on publicly available patent literature, which may overlook some proprietary technologies or undisclosed research outcomes, leading to an incomplete understanding of the overall landscape of technology evolution. Furthermore, although this study explores technological dependencies and control patterns, it fails to delve deeply into the long-term impacts of regional differences in technological innovation capabilities and industrial policies on technological evolution. Therefore, future research should consider utilizing more diverse data sources, such as industry reports, corporate financial data, and expert interviews, to provide a more comprehensive insight into the driving factors behind technological evolution. Additionally, an interdisciplinary perspective that incorporates theoretical frameworks from fields like economics and sociology would contribute to a deeper understanding of the innovation ecosystem within the chip sector. Finally, future research should also prioritize how to foster cooperation and the sharing of technologies to reduce barriers and promote sustainable development in the global chip industry. By addressing these limitations and outlining future research directions, we aim to lay a foundation for further studies that may facilitate comprehensive advancements in technology and industrial innovation.

### 4.2. Recommendations

#### 4.2.1. R&D Resource Allocation and Technology Pathway Planning

Based on core technology entropy evolution analysis, this study recommends establishing a dynamic resource allocation mechanism to adapt to the cyclical characteristics of technological innovation. The nonlinear fluctuation trajectory of timing-aware manufacturability-driven collaborative routing technology (3.91→2.81→3.58) indicates that R&D investment should follow a cyclical pattern of “increased investment during high-entropy phases—maintenance during low-entropy phases—renewed investment during paradigm reconstruction periods.” In response to the phenomenon of 3D stacking packaging technology entropy increasing from 6.41 to 8.68, R&D planning should prioritize the deployment of multi-physics coupling simulation platforms and heterogeneous material integration technologies, achieving technological convergence innovation through interdisciplinary project portfolios. Regarding regional innovation ecosystem positioning, China’s breakthrough in angular filtering rotation OPC methods (entropy reduction to 3) demonstrates the effectiveness of focusing on specific technology nodes. This study recommends adopting a “point breakthrough—chain expansion” strategy, achieving competitive advantages through specialized deep cultivation within globally dominant technology domains. Simultaneously, attention should be paid to the specialized value of low-entropy technology nodes, constructing sustainable competitive advantages through deep vertical domain iteration while avoiding the blind pursuit of technological coverage breadth.

#### 4.2.2. Policy Framework Design and Risk Management

Technology dependency risk assessment should establish differentiated policy frameworks based on the spatial differentiation characteristics of patent layouts. The contrast between upstream patent globalization layout and downstream patent regionalization application revealed by ALD equipment technology pathways suggests that policy formulation should distinguish between fundamental process innovation dependencies and application optimization opportunities, implementing strategic technology reserve policies for the former while encouraging international cooperation for the latter. The phenomenon of EUV lithography supporting material gaps propagating through multilateral intellectual property systems emphasizes the importance of selective technology absorption policies. This study recommends establishing a progressive technology acquisition mechanism of “open introduction—digestion and absorption—re-innovation.” Innovation ecosystem architecture design should integrate entropy theory monitoring systems, utilizing information entropy indicators as early warning signals for technology paradigm shifts to transform policy adjustments from passive responses to proactive anticipation. The staged growth in entropy values of pitch isolation technology (1.1→1.8) demonstrates a “technology introduction → independent innovation” transformation model, providing an empirical template for constructing phased innovation policies. Policy frameworks should establish entropy threshold indicators, triggering independent innovation support mechanisms when entropy values in technology domains show sustained increases.

#### 4.2.3. Investment Strategy Optimization and Industrial Coordination

Investment timing optimization should establish a quantitative decision-making framework based on entropy evolution patterns. High-entropy phases (>6.0) correspond to fundamental breakthrough opportunities, suitable for concentrated venture capital deployment, while low-entropy phases (<3.0) represent engineering optimization opportunities, appropriate for stable development funding support. The centralization trend of 3D stacking packaging technology (entropy decline accompanied by patent layout concentration toward the United States) suggests that investment strategies should establish phased exit mechanisms, capturing technology value during expansion periods (high entropy, dispersed layout) and realizing financial returns during consolidation periods (low entropy, concentrated layout). Strategic technology acquisition and cooperation should focus on technology bridging nodes. The phenomenon of high-entropy patents connecting different technology subcategories in flip-chip packaging evolution indicates that enterprises positioned at technology convergence points often possess platform value, providing multi-pathway technology integration opportunities. The pattern of South Korea–US bilateral citation intensity, accounting for 58% of cross-border citations, reveals the technological complementarity in equipment–materials collaborative development. Investment strategies should prioritize transnational cooperation projects that can leverage such complementarity. Long-term strategic planning should establish entropy-oriented technology roadmaps, utilizing cyclical entropy fluctuations as criteria for judging technology life cycles. Through the cyclical model of “high-entropy exploration—low-entropy convergence—renewed high-entropy breakthrough,” industrial investment rhythms should be guided to ensure forward-looking deployment capabilities during technology paradigm transition periods.

## 5. Conclusions

### 5.1. Identification of Core Technologies and Summary of Technical Path Evolution in the Chip Domain

Within the chip design phase, five core technologies exhibit profound cross-domain integration. Circuit layout simulation technology, for instance, demonstrates patent concentration in G06F017/50 (computer-aided design) and H01L021/00 (semiconductor device manufacturing), with a discernible diffusion toward G05F001/59 (circuit power control) and G06F021/00 (data security technology). Optical Proximity Correction (OPC) technology anchors its technical foundation in G03F007/20 (optical lithography processes) and G03F001/14 (lithography mask design), with complexity escalating through penetration into G03F001/78 (mask defect correction). The evolution of 3D chip stacking Through-Silicon Via (TSV) interconnection technology, meanwhile, extends from foundational processes like wafer thinning and metallization across over 20 subfields, embodying the multi-physical-field coupling complexity inherent to advanced design.

Focusing on the chip manufacturing phase, process deepening defines the four core technological advancements. Thin-film microstructure etching, built upon traditional semiconductor processes, progresses toward high aspect ratio structure processing and 3D device compatibility. High-κ metal gate integration relies heavily on the atomic layer deposition (ALD) framework, with patent classifications clustering at the intersection of C23C (coating technology) and H01L (semiconductor devices). Interlevel isolation technology, starting from basic classifications, such as H01L021/334 and H01L029/768, evolves toward precision-oriented domains, like H01L021/306 (high aspect ratio structure processing) and H01L023/522 (interconnection layer isolation), reflecting intensified process control requirements.

Shifting focus to the packaging and testing domain, integration and intelligence characterize the five core technologies. Wafer-level packaging expands its IPC classification from the baseline H01L021/02 wafer bonding technology across 25 specialized subdirections, showcasing modular innovation. Flip-chip packaging undergoes a material technology transition—from B32B027/38 (laminated structure adhesive layers) to C09J175/14 (polyurethane adhesives)—signifying incremental material science advancements. Built-in self-test technology, importantly, evolves from G01R31/3185 (scan chain testing) to G06F11/27 (self-checking fault-tolerant systems), marking a pivotal shift from isolated functional testing to holistic system-level reliability management.

### 5.2. Identification of Emerging Trends and Evolution Patterns

Building upon the core technology identification framework established above, this study further reveals the emerging trend characteristics of technological evolution in the semiconductor industry.

(1) Evolution Characteristics of Technology Convergence Trends

Based on entropy-based analysis methods, this research identifies three significant cross-domain technology convergence directions within the semiconductor industry. First, the design and manufacturing stages demonstrate a collaborative optimization convergence trend. Taking timing-aware manufacturability-driven collaborative routing technology as an example, its entropy evolution trajectory (3.91→2.81→3.58) clearly illustrates a complete technological evolution cycle from theoretical breakthrough to engineering convergence, and then to new paradigm reconstruction. This nonlinear variation pattern reflects the technological iteration patterns inherent in the design-manufacturing integration process. Second, the packaging and testing stages exhibit an intelligent integration development trend. The substantial increase in entropy values for 3D stacking packaging technology from 6.41 to 8.68 signifies that this field is undergoing a critical technological transition from adhesive-free bonding toward inorganic material-oriented convergence, embodying the intrinsic logic of packaging technology evolution toward high integration density and high reliability.

(2) Differentiation Pattern of Regional Innovation Ecosystems

Spatial analysis of patent landscape reveals that the global semiconductor industry exhibits a knowledge flow network structure characterized by “US origination—East Asian implementation—multi-center improvement.” In terms of technological innovation capability, China has achieved breakthrough innovations at specific technological nodes, where the entropy reduction to 3 for the angular filtering rotation OPC method (CN112462570-A) indicates that China has realized deep breakthroughs in specialized technical fields such as mask geometry correction, demonstrating positive signals of transition from technology following to technology leading. Regarding international collaboration networks, the bilateral citation intensity between South Korea and the United States reaches the highest level, accounting for 58% of total cross-border technology citations. This data fully highlights the technological complementarity and strategic cooperation depth between the two countries in equipment-materials collaborative development, reflecting the characteristics of the international division of labor in technology-intensive segments of the global semiconductor industry chain.

### 5.3. Technology Gap Identification and Control Pattern Analysis

Despite the positive trends of technology convergence and innovation ecosystem differentiation in the semiconductor industry, in-depth technology gap analysis reveals that significant technological dependencies and control centralization issues persist in critical domains.

(1) Technological Dependency Characteristics in High-End Process Equipment

Deep analysis of ALD (ALD—Atomic Layer Deposition—a thin film deposition technique that deposits materials one atomic layer at a time through sequential, self-limiting chemical reactions, providing excellent conformality and thickness control for 3D structures) equipment technology pathways reveals significant technological dependency patterns in the global semiconductor industry. From the patent landscape dimension, upstream fundamental innovation patents are primarily deployed globally through international channels such as WO (World Intellectual Property Organization), US (United States), JP (Japan), and KR (South Korea), while downstream application patents exhibit distinct regionalization characteristics, mainly concentrated in specific markets, such as CN (China). This stark contrast reflects the specialized transfer trend of technical knowledge from fundamental process innovation to specific performance parameter improvements in the semiconductor industry. In critical technology domains, the technological gap in extreme ultraviolet (EUV) lithography supporting materials is particularly prominent, specifically manifested as high-entropy patents in the optical proximity correction technology evolution pathway continuously propagating and diffusing through multilateral intellectual property systems, such as WO and EP (European Patent Office), while regional patents from CN and TW (Taiwan) primarily focus on process detail optimization, objectively reinforcing the construction of local technological barriers.

(2) Control Pattern Analysis of Advanced Packaging Technologies

Evolution trajectory analysis of 3D stacking packaging technology indicates a clear trend toward technological control centralization in this field. From the patent citation network perspective, the concentrated citation pattern of US patents suggests continuous strengthening of leading enterprises’ control capabilities over high-density integration technologies, reflecting the increasingly elevated technological thresholds and market entry barriers in the advanced packaging field. From the technological complexity evolution perspective, the peak in technological complexity that emerged in 2020 precisely corresponds to the critical temporal node of frequent cross-citations among multiple technology nodes, marking an important inflection point for technological integration in this field. Notably, with the gradual decline in entropy values, the technology citation network exhibits a significant trend of transformation from cross-border multi-directional flow patterns toward regional closed-loop patterns. This change reveals that the global semiconductor industry chain is undergoing a complex evolution process characterized by the coexistence of technological standardization, convergence, and regionalized division of labor in the advanced packaging field.

## Figures and Tables

**Table 1:** Chip design LDA core topic words.

Clustering Results	Core Topic	LDA Topic Words
Cluster 0	Topic 7	Clock_Signal_Path; Dynamically_Update_Timing_State; Aggregate_Rout_Cost; Multiple_Rout_Abstraction_Level; Metal_Line_Failure
Cluster 1	Topic 4	Voltage_Regulator; Logic_Cell; Boolean_Logic_Cell; Power_Definition; Data_Character_Precoding_Correction
Cluster 2	Topic 2	Microstrip_Bandpass_Filter_Module; Convex_Portion; Line_Pattern_Deformation; CMOS_Structure; Parasitic_Capacitance_Difference
Cluster 3	Topic 3	Low_Leakage_Power_Optimization; Activity_Factor; Hvt_Cell; Power_Estimation_Tool; Critical_Path_Determination
Cluster 4	Topic 7	Stack_Circuit; Dice_Vias; Create_Interposer; Semiconductor_Chip_Portion; Physical_Structure

**Table 2:** Core technologies and technical descriptions in chip design.

Technology Topic	Technical Description
Circuit Layout and Simulation Flowchart	A design methodology encompassing the complete workflow from circuit schematic to physical layout, including logic synthesis, placement and routing, timing analysis, and verification processes
Timing Manufacturability Collaborative Routing Optimization Technology	An optimization framework that simultaneously addresses timing constraints and manufacturing limitations during routing, ensuring designs meet performance and manufacturability requirements
Optical Proximity Correction Technology	A computational lithography technique that compensates for optical diffraction effects during photolithography by pre-distorting mask patterns to ensure accurate pattern transfer to silicon
Intelligent Power Optimization Technology	An AI-driven power management approach using machine learning algorithms for power analysis and optimization, incorporating power gating, clock gating, and voltage scaling to minimize consumption
Silicon Via Interconnection Technology for 3D Chip Stacking	A technology enabling vertical electrical connectivity between stacked die layers through through-silicon vias (TSVs), facilitating enhanced integration density and improved system performance

**Table 3:** Chip manufacturing LDA core topic words.

Clustering Results	Core Topic	LDA Topic Words
Cluster 0	Cluster 0	Dry_Etch_Method; Photo_Etch_Prcess; Bhin_Film_Remove_Method; Chemical_Mechanical_Process; Wet_Etch_Process
Cluster 1	Cluster 1	Micro_Scale_Recess_Structure; Micro_Scale_Protrude_Structure; Medium_Substrate; Pole_Isolation; Photolithography_Mask_Alignment
Cluster 2	Topic 10	High_K_Metal_Gate; BEOL_Wiring; Low_K Dielectric_Materials; High_Electron_Mobility; Low_K_Bonding_Layer
Cluster 3	Topic 8	Thin_Film_Deposit_Apparatus; Vapor_Deposition_Material; Thin_Film_Transistor_Tube; Epitaxial_Growth; Nanowire_Growth_Condition

**Table 4:** Core technologies and technical descriptions in chip manufacturing.

Technology Topic	Technical Description
Thin Film Microstructure Etching Technology	A precision fabrication technique for creating well-defined microstructures in thin films through controlled material removal processes, utilizing plasma etching, reactive ion etching, or wet chemical etching to achieve high aspect ratios and dimensional accuracy
Inter Electrode Isolation Technology	A process technology designed to prevent electrical interference and crosstalk between adjacent electrodes through the implementation of isolation barriers, dielectric layers, or physical separation structures to maintain signal integrity
High Dielectric Constant Metal Gate (HKMG) Integration Technology	An advanced gate stack technology that replaces traditional polysilicon gates with metal electrodes and high-k dielectric materials to reduce gate leakage current, improve drive current, and enable continued transistor scaling in nanoscale devices
Atomic Layer Deposition (ALD) Technology	A thin film deposition technique that enables precise thickness control at the atomic level through sequential, self-limiting surface reactions, providing excellent conformality and uniformity for coating complex three-dimensional structures

**Table 5:** Core keywords for chip packaging and testing LDA.

Clustering Results	Core Topic	LDA Topic Words
Cluster 0	Topic 3	Flip_Chip_Epitaxial_Wafer; Wafer_Level_Packaging_Structure; Wafer_Level_Packaging_Method; Hermetic_Type_Chip; Packaging_Stability
Cluster 1	Cluster 1	Flip_Chip_Package_Structure; Undfill_Process; Anisotropic_Conductive_Adhesive; Wire_Bonding_Process; Solder_Mask_Layer
Cluster 2	Topic 3&6	Stack_Package; nterconnect_Metal_Trace; Fan_Wafer_Level_Packaging; Through_Silicon_Vias; Die_Stack_Process
Cluster 3	Topic 4	Built_Self_Test; Bist_Engine; Test_Operation; Scan_Test; Test_Wrapper
Cluster 4	Topic 7	Contact_Pin; Test_Head; Test_Apparatus; Test_Point_Site; Probe_Device

**Table 6:** Core technologies and technical descriptions in chip packaging and testing.

Technology Topic	Technical Description
Wafer Level Packaging Technology	A packaging approach that performs encapsulation and interconnection at the wafer level before die separation, enabling miniaturization and cost reduction
Flip Chip Packaging Technology	A direct chip attachment method using solder bumps to create electrical connections between die and substrate, eliminating wire bonds for high-density I/O
Optical Proximity Correction Technology	A vertical integration technique that stacks multiple dies or packages to achieve higher density, reduced footprint, and enhanced performance
Intelligent Power Optimization Technology	An electrical testing method using precision probes to contact wafer pads for functional verification before packaging, enabling early defect detection
Silicon Via Interconnection Technology for 3D Chip Stacking	An on-chip testing approach incorporating dedicated test circuits for autonomous verification, reducing external test equipment requirements

**Table 7:** Topic coherence comparison experiment.

	Chip Design		Chip Manufacturing		Chip Packaging and Testing	
Coherence	U_Mass	C_V	U_Mass	C_V	U_Mass	C_V
PKCN-BERT-LDA	−2.01	0.51	−5.57	0.56	−1.25	0.55
BERT	−3.68	0.38	−5.69	0.48	−4.66	0.40
Word2Vec	−3.76	0.47	−5.60	0.51	−2.00	0.57
LDA	−8.50	0.42	−7.76	0.38	−7.52	0.36

## Data Availability

The original data presented in this study are openly available in [Derwent Innovations Index] at [https://www.webofscience.com/wos/diidw/basic-search (accessed on 2 January 2025)].
